# CYP5122A1, a Novel Cytochrome P450 Is Essential for Survival of *Leishmania donovani*

**DOI:** 10.1371/journal.pone.0025273

**Published:** 2011-09-23

**Authors:** Smriti Verma, Ashish Mehta, Chandrima Shaha

**Affiliations:** Cell Death and Differentiation Research Laboratory, Centre for Molecular Medicine National Institute of Immunology, New Delhi, India; Federal University of São Paulo, Brazil

## Abstract

**Background:**

Cytochrome P450s (CYP450s) are hemoproteins catalysing diverse biochemical reactions important for metabolism of xenobiotics and synthesis of physiologically important compounds such as sterols. Therefore, they are functionally important for survival of invading pathogens. One such opportunistic pathogen *Leishmania donovani* causes visceral leishmaniasis worldwide, which is an important public health problem due to significant disease burden. The parasite genome database, Gene DB, annotates 3 CYP450s in *Leishmania*, however, the functional role of cytochrome P450 enzymes in *Leishmania* spp. remains elusive.

**Methodology/Principal Findings:**

A CYP450-like gene cloned from *Leishmania donovani* was identified as a novel CYP450, the CYP5122A1. Upon co-localization with organelle specific markers, CYP5122A1 distribution was shown to be localized in the promastigote ER, mitochondria and the glycosomes. Replacement of one allele of CYP5122A1 with either neomycin or hygromycin gene by homologous recombination in *Leishmania* promastigotes induced substantial reduction of CYP5122A1 expression. These parasites showed impaired growth, lower mitochondrial Ca^2+^ and membrane potential resulting in low ATP generation. Also, these parasites were less infective *in vitro* and *in vivo* than their wild-type counterparts as assessed by incubation of *Leishmania* promastigotes with macrophages *in vitro* as well as through administration of parasites into hamsters. The HKOs were more susceptible to drugs like miltefosine and antimony, but showed reduced sensitivity to amphotericin B. Removal of two alleles of CYP5122A1 did not allow the parasites to survive. The mutant parasites showed 3.5 times lower ergosterol level as compared to the wild-type parasites when estimated by Gas chromatography/mass spectrometry. Complementation of CYP5122A1 through episomal expression of protein by using pXG-GFP+2 vector partially rescued CYP5122A1 expression and restored ergosterol levels by 1.8 times. Phenotype reversal included restored growth pattern and lesser drug susceptibility.

**Conclusions/Significance:**

In summary, this study establishes CYP5122A1 as an important molecule linked to processes like cell growth, infection and ergosterol biosynthesis in *Leishmania donovani*.

## Introduction

For survival within host cells, parasites are required to eliminate host generated oxidative stress products, maintain biosynthesis of essential molecules and resist lethal drugs. *Leishmania donovani*, a kinetoplastid parasite, causes visceral leishmaniasis (VL) in humans and the disease remains a significant problem in the tropics due to emergence of drug resistance [1]. This situation is compounded by overlap of endemic regions of VL with regions of HIV infection where the opportunistic pathogen *Leishmania* presents a major problem [2]. *Leishmania* parasites have a digenetic life cycle where infectious metacyclic promastigotes differentiate within the sand fly and following transfer to the mammalian host through sandfly bite, the parasites thrive as non-motile amastigotes within the macrophages [1], [3] Earlier studies from this laboratory and others have shown that both the promastigote and the amastigote forms of *Leishmania donovani* are sensitive to oxidative and nitrosative stress and use specialized defense systems unique to themselves to survive when exposed to such conditions [4]–[7]. In the absence of an effective vaccine, limited chemotherapeutic drugs and growing drug resistance, it has become important to increase the understanding of the physiology of the *Leishmania* parasite to explore for new drug targets. Although several aspects of *Leishmania* cellular defense are known, the role of CYP450-like proteins in the survival of this parasite remains to be explored.

CYP450s are hemoproteins catalysing a variety of chemical reactions including biotransformation of drugs, bioconversion of xenobiotics, chemical carcinogen metabolism and the synthesis of physiologically important compounds such as sterols and fatty acids [8]. A clear indication of CYP450 involvement in *Leishmania* survival comes from studies where CYP450 inhibitors, the azole antifungals namely itraconazole, ketoconazole and fluconazole, have been used as successful antileishmanial agents [9]. Azole antifungals also cause radical parasitological cure in murine models of Chagas' disease, caused by a related parasite, the *Trypanosoma cruzi* through inhibition of CYP51, a sterol C14 alpha-demethylase [9]–[10]. Reportedly, *Leishmania* cell lysates catalyse CYP450-like reactions [11]. The above studies clearly indicate a functional role of CYP450s in kinetoplastid parasite biology; however, no reports are available on the actual functional involvement of these molecules. Due to their involvement in multiple functional systems, CYP450s are potential drug targets as shown in *Mycobacterium tuberculosis* [12] and are reportedly associated with drug resistance in *Candida albicans* [13].

One of the important functions of CYP450s is biosynthesis of ergosterol [14] which is the primary component of the *Leishmania* membrane and is functionally linked to maintenance of structural integrity and protection from biotic stress [15]. Therefore, interference with ergosterol biosynthesis could result in disruption of parasite function and molecules involved in this pathway could serve as potential drug targets [16]. Interestingly, *Leishmania* can survive altered sterol levels [17] but changes in sterol profile have been linked to amphotericin B and fluconazole resistance [9], [18]. The genome database of the kinetoplastid parasites annotate three putative CYP450-like proteins amongst the members of the genus *Leishmania* and *Trypanosoma*, however, functional information on none of the three CYP450-like proteins is available which could have important role in *Leishmania* survival related to drug response, synthesis of biologically important molecules and elimination of xenobiotics. Therefore, it is important to analyse the function of *Leishmania* CYP450s to understand multiple functional aspects of parasite physiology that contribute to cell survival and drug resistance.

In this study, we identify CYP5122A1 as a novel CYP450 of *Leishmania donovani* playing a role in several important events in parasite function including response to drugs and the ability to infect.

## Results

### CYP5122A1 is well conserved within *Leishmania* spp

To probe into the function of one of the CYP450-like proteins in *Leishmania donovani*, the gene was cloned using primers designed from the sequence of its orthologue in *L. major* (Lmj F27.0090, Gene DB, Wellcome Trust Sanger Institute, Hinxton, UK). The sequence of the full length clone (Acc. No. DQ267494) was classified by the ‘Cytochrome P450 Nomenclature Committee’ [19] as CYP5122A1 (Nelson D.R. at The Cytochrome P450 Homepage, http://drnelson.uthsc.edu/CytochromeP450.html; personal communication). The CYP5122A1 sequence shared close identities with CYP450–like proteins of other members of the *Leishmania* spp. like *Leishmania major*, *Leishmania infantum*, *Leishmania mexicana*, *Leishmania braziliensis* (87–98%) as well as with the *Trypanosoma* spp. (50–53%). A phylogenetic tree comparing CYP5122A1 with CYP450s from other species show *Leishmania* CYP450s as a separate cluster from *Trypanosoma* which is the closest neighbour, the evolutionary distance from mammals being large (Fig. S1).

**Figure 1:**
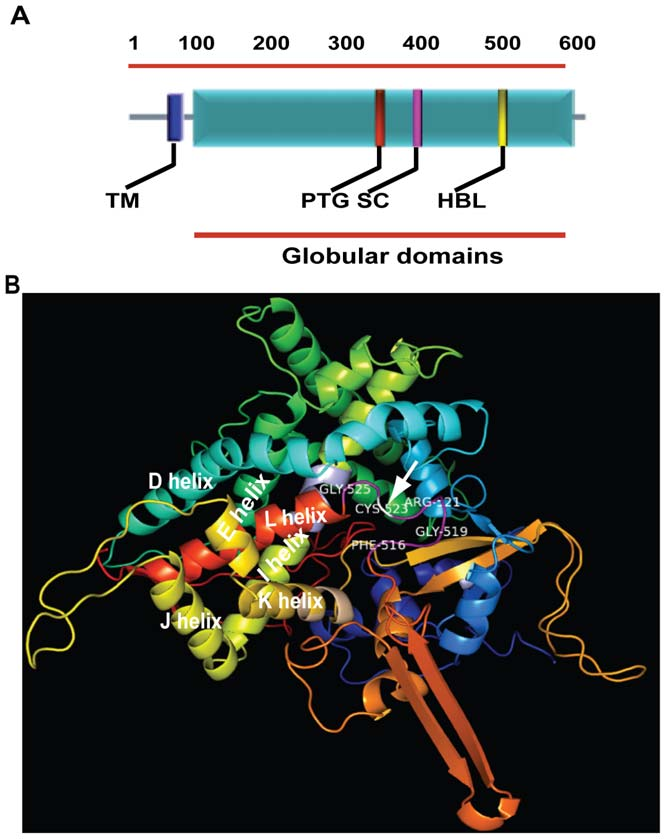
Structural analysis of CYP5122A1. **A:** Protein sequence based schematic representation of CYP450 conserved motifs in CYP5122A1 sequence. TM, Trans-membrane domain; PTG, Proton transfer groove; SC, Stabilization core; HBL, Heme binding loop. **B:** A ribbon representation of folded CYP5122A1 protein structure generated using SWISS-MODEL server. α-helix, coiled ribbon; β-sheet, arrow; random coil and loop, line. The motifs shown are, stabilization of the core (pink) in the K helix; the proton transfer groove in central part of the I-helix, mauve; heme binding loop, purple; conserved Cys (523), indicated by an arrow.

A characteristic feature of P450 superfamily is a high conservation in their general topology and structural fold despite low sequence similarity; however, no two CYP450s are structurally identical [20]. The CYP5122A1 sequence showed the presence of a transmembrane domain (TM; amino acids 38–60), a proton transfer groove (PTC; amino acid 348–353), a motif for stabilization of the core (SC; amino acid 418–421) and a heme binding loop (HBL; amino acid 516–525) (Fig. 1A) that are characteristics of CYP450 proteins. Homology modelling attempts for CYP5122A1 on the RCSB (Research Collaboratory for Structural bioinformatics) PDB (www.pdb.org/) server identified several templates of CYP102 (BM-3) usable for homology modelling of CYP5122A1. One such template 1ZOA generated a model of CYP5122A1 spanning from 70 to 590 of 592 residues including the active site heme binding motif containing the characteristic P450 consensus sequence represented by Phe-Ile-Asn-Gly-Pro-Arg-Asn-Cys-Leu-Gly (amino acids 516 to 525). The conserved catalytic cysteine (marked by arrow), a ligand for the heme iron, occupies position 523 (Fig. 1B, Fig. S2), in the heme binding loop (Fig. 1A), and is exposed in a pocket just before the L helix (Fig. 1B; Fig. S2), as is observed in all the cytochrome P450s in general. The conserved motif for stability of the CYP450s (SC; Fig. 1A) was represented by Glu-Thr-Leu-Arg (amino acids 418 to 421; Fig. S2) located in the helix K on the proximal side of the heme binding domain (Fig. 1B, Fig. S2). The proton transfer groove (PTG; Fig. 1A) containing amino acids Ala-Gly-His-Glu-Thr-Ser (amino acids 348 to 353; Fig. S2), located on the distal side of the heme in the central part of I-helix was close to the substrate binding site (Fig. 1B). The overall packing quality of the model assessed using WHATCHECK server was within normal range (z = −1.329). Further analysis of the Ramachandran plot of the same generated by PROCHECK, showed majority (85%) of amino acids in the highly favourable region (Fig. S3). Therefore, the above data shows that CYP5122A1 of *L. donovani* contains CYP450 consensus sequences and shares close identity with CYP450 proteins of both the old and the new world *Leishmania* spp.

### CYP5122A1 shows no major change during differentiation of metacyclics and is located at different subcellular locations

**Figure 2:**
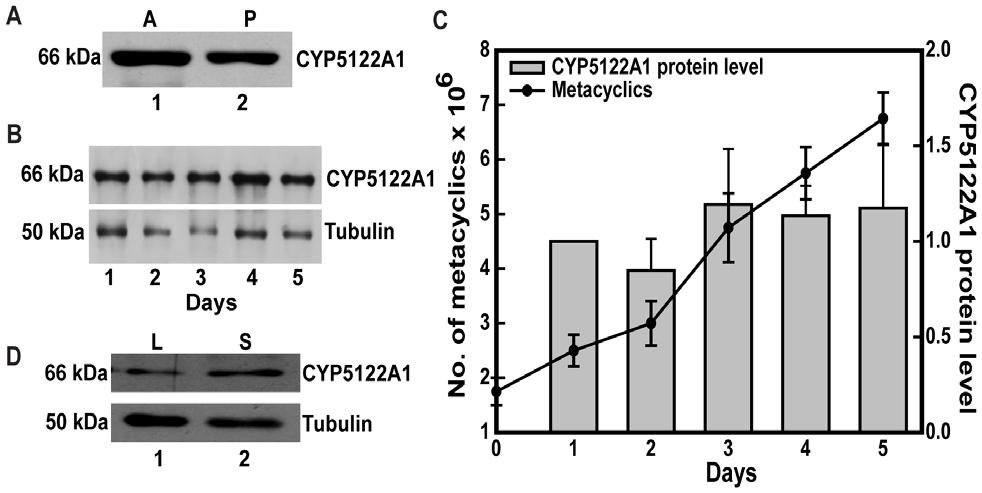
Expression of CYP5122A1 in amastigotes and promastigotes of *L. donovani*. **A:** Western blot analysis of equal protein quantities from whole cell lysates prepared from *L. donovani* amastigotes (A) and promastigotes (P) probed with anti-CYP5122A1 antibody. **B:** Western blots of total cell lysates prepared from *L. donovani* promastigotes at different time points during an *in vitro* growth cycle, probed with anti-CYP5122A1 antibody. Loading was normalized with Tubulin–α (50 kDa). **C:** Bar graph; right hand y-axis represents quantitation of immunoblots shown in B, mean ± SE, n = 3. Line graph, left hand y-axis represents the number of metacyclic parasites formed during the *in vitro* growth cycle. **D:** Western blot analysis of whole cell lysates prepared from *L. donovani* metacyclic prepared from logarithmic (L) and stationary (S) phase culture probed with anti-CYP5122A1 antibody indicating marginal up-regulation of CYP5122A1.

Parasite growth and differentiation into infective forms (metacyclics) within the insect host can be mimicked *in vitro* by culturing flagellated promastigotes [21]. To check intracellular levels of CYP5122A1, a specific antibody raised against the most immunodominant sequence stretch on CYP5122A1 that recognized a single band in *Leishmania donovani* whole cell lysates was used. When the presence of CYP5122A1 in the promastigotes and the amastigotes was checked, both forms showed the presence of CYP5122A1, where the total concentration of the protein appeared to be higher in the amastigotes (Fig. 2A). As shown in Fig. 2B, no major change in the intracellular CYP5122A1 levels was observed during differentiation of the metacyclics with the constitutive levels remaining fairly constant in an unsynchronized culture (Fig. 2C, bar graph). However, when we looked at protein levels in metacyclic parasites from logarithmic (day 3) and stationary phase (day 5), a higher CYP5122A1 protein level was observed in the metacyclics isolated from stationary phase cultures (Fig. 2D, lane 2) in comparison to those isolated from log phase cultures (Fig. 2D, lane 1).

**Figure 3:**
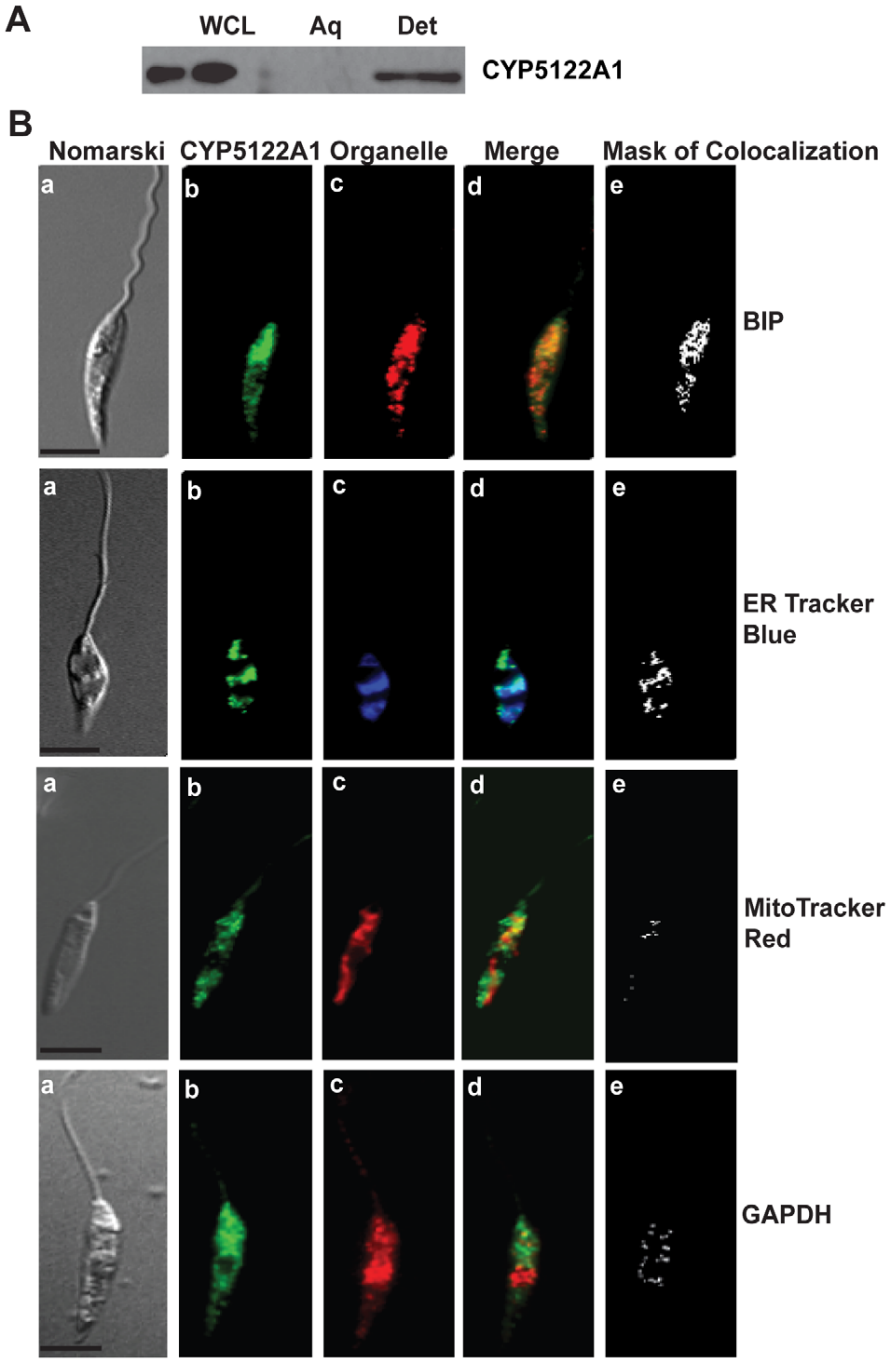
Subcellular localization of CYP5122A1. **A:** Western blot of aqueous (Aq) and detergent (Det) phases of Triton X-114 extracts of *Leishmania* promastigotes probed with anti-CYP5122A1 antibody. **B:** Photomicrographs of *Leishmania donovani* promastigotes stained with anti-CYP5122A1 antibody and organelle specific markers. BIP & ER Tracker Blue-White DPX, ER marker; MitoTracker Red, mitochondrial marker; GAPDH, glycosome marker. **a**: Nomarski; **b**: cells stained with anti-CYP5122A1 antibody; **c**: cells stained for organelle specific marker; **d**: merge of b and c; **e**: mask of colocalization for d. Scale represents 10 µm.

CYP5122A1 was associated with the lipid fraction and not the aqueous fraction of cell extracts prepared with Triton-X114 (Fig. 3A) suggesting membrane association of the protein which is in concurrence with the presence of a membrane spanning domain between amino acids 38–60 (Fig. 1A). Promastigotes from log phase cultures stained with anti-CYP5122A1 and counter-stained with BIP, an hsp70 family protein found in ER, and ER Tracker Blue®, a dapoxyl dye that stains ER in live cells, showed co-localization of ER and CYP5122A1 stain indicating localization of the protein to the ER (Fig. 3B, a–e, BIP and ER Tracker blue). In addition, although less predominant, co-localization of CYP5122A1 staining was also seen in the mitochondria identified using MitoTracker Red® or glycosomes marked with glyceraldehyde-3-phosphate dehydrogenase (GAPDH) (Fig. 3B, a–e, MitoTracker red and GAPDH). Therefore, these observations show that CYP5122A1 distribution is confined predominantly to the endoplasmic reticulum and in smaller amounts to the mitochondria and the glycosomes. The metacyclic forms of the parasite were isolated and stained with anti-CYP5122A1 antibody and anti-BIP antibody to see if the staining pattern was similar to log phase promastigotes. Co-localization of CYP5122A1 with BIP was observed indicating that there was no apparent difference in the localization of the protein as cells differentiated into metacyclics (Fig. S7).

### CYP5122A1 is essential for optimal growth of the parasite

**Figure 4:**
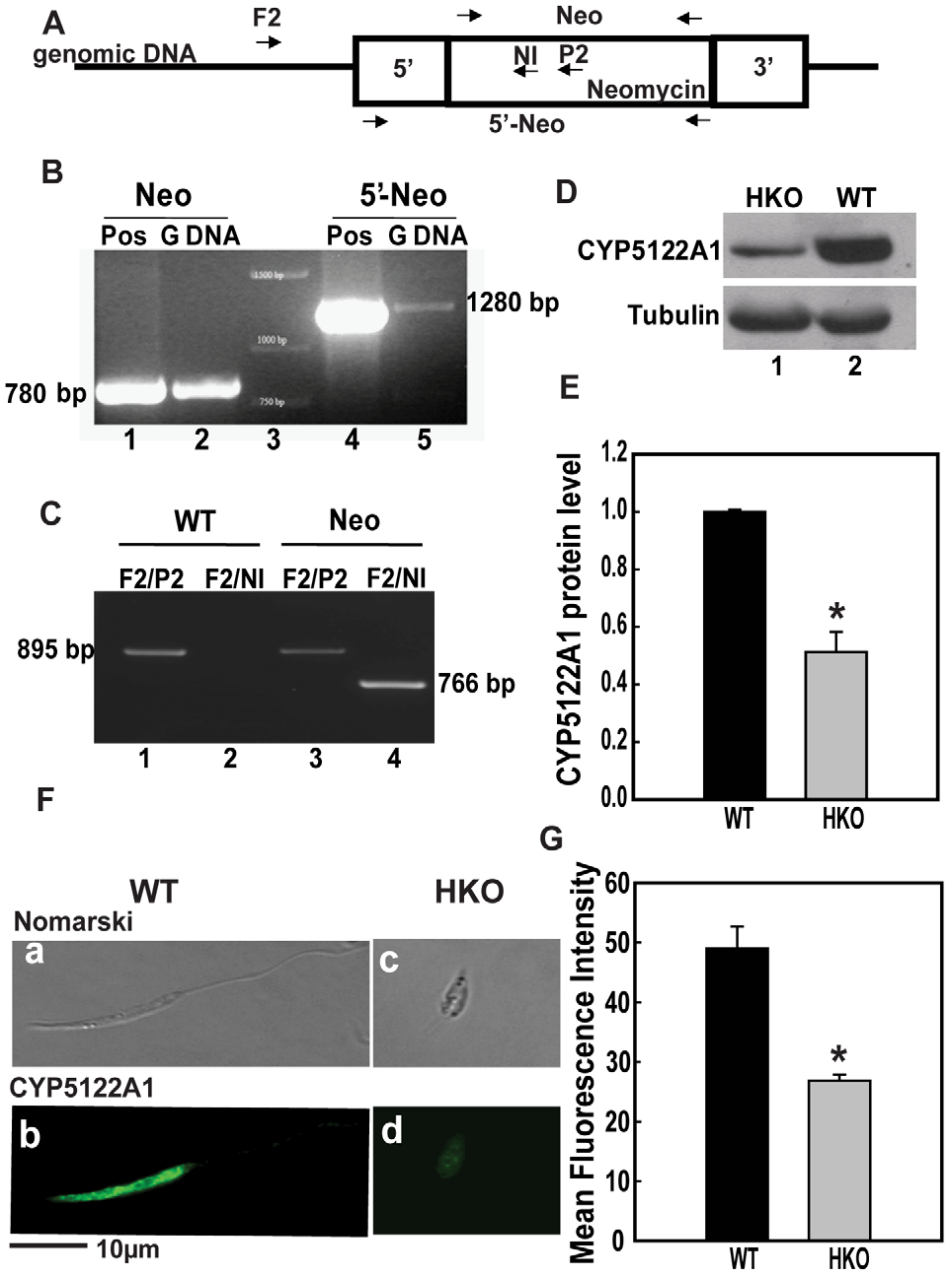
Generation of CYP5122A1 half knock-outs. **A:** Schematic representation of replacement construct inserted into the genome with positions of primers used for confirmation marked by arrows. 5′: 5′ homologous sequence; 3′: 3′ homologous sequence; Neomycin: ORF encoding for resistance to neomycin; F2: forward primer designed in the intergenic region upstream of CYP5122A1 gene; P2: internal primer in CYP5122A1 encoding sequence that has been replaced in the replacement construct; NI: primer designed in the internal sequence of ORF for neomycin resistance; Neo: primer pair amplifying neomycin resistance ORF, 5′-Neo: Primers that amplify sequence spanning the 5′ homologous region through to the end of neomycin resistance ORF. **B:** Insertion of allelic replacement construct was confirmed by PCR for neomycin and knockout-neomycin construct (5′-Neo) using primers that span the 5′ homologous sequence (Forward primer) and the neomycin gene (Reverse primer). Lane 1: positive control (Pos) for neomycin resistance gene (plasmid); Lane 2: fragment obtained after amplification of neomycin resistance gene from genomic DNA (G DNA); Lane 3: 1 kb DNA Ladder; Lane 4: Positive control for knock out–neomycin construct (plasmid); Lane 5, Fragment amplified for the knockout-neomycin construct. **C:** Confirmation of insertion of the replacement construct at the correct locus was assessed by PCR. Lane 1: amplicons generated from WT genomic DNA as template with primers F2/P2 indicating the presence of intact CYP5122A1 allele; Lane 2: Primers F2/NI did not generate any amplicons with WT genomic DNA as template, showing the absence of neomycin resistance ORF; Lane 3: amplicons generated from HKO genomic DNA as template with primers F2/P2 indicating the presence of an intact CYP5122A1 allele; Lane 4: amplicons generated from HKO genomic DNA as template with primers F2/NI indicating the presence of insertion of neomycin resistance ORF at the correct locus. **D:** Western blot analysis of cell lysates from WT and HKO parasites probed with anti-CYP5122A1 antibody showing decreased level of CYP5122A1 protein in the HKO parasites. Loading was normalized with Tubulin–α (50 kDa). **E:** Bar graph representing averaged densitometric analysis of immunoblots represented in D (mean± SE, n = 3), * P≤0.05. **F:** Photomicrographs of *L. donovani* WT (a, b) and HKO (c, d) parasites immunostained with anti-CYP5122A1. Scale, 10 µm. **G:** Bar graph representing mean fluorescence intensities of CYP5122A1 fluorescence in WT versus HKO parasites (mean± SE, n = 3), * P≤0.05.

To determine the importance of CYP5122A1 in the survival of *Leishmania donovani*, the CYP5122A1 encoding sequence was disrupted by ORFs encoding for neomycin or hygromycin resistance. Gene replacement vectors (pBSK+CYP5122A1Neo or pBSK+CYP5122A1Hyg) were constructed such that ORFs encoding neomycin/hygromycin resistance were flanked by sequences homologous to 5′ and 3′ region of the target gene. These vectors were used to transform wild-type (WT) cells where homologous recombination resulted in one allele being replaced by the antibiotic resistance gene and the resultant parasites had only one functional allele of CYP5122A1 (half knockout, HKO). These parasites with one allele of CYP5122A1 were re-transfected with the vectors containing the second antibiotic resistance gene to obtain replacement of the remaining allele so that parasites expressing no CYP5122A1 could be obtained. These double knockout parasites did not survive in culture suggesting that the gene may be essential for parasite survival. Therefore, we used the HKOs containing one allele of CYP5122A1 for further functional experiments. For characterization of these HKO parasites, the successful deletion one WT copy of the CYP5122A1 gene was confirmed by PCR with genomic DNA of the HKOs using primers (primer positions marked in the schematic, Fig. 4A) for neomycin (Fig. 4B, lane 2), as well as a forward primer spanning the 5′ end of the CYP5122A1 gene and reverse primer for the neomycin (Fig. 4B, lane 5) or hygromycin ORF (Fig. S4A (i), lane 1) and 5′-hygromycin fragment (Fig. S4A (i), lane 3). This confirmed the presence of the neomycin/hygromycin resistance gene. To confirm the correct targeting of the replacement construct to the CYP5122A1 locus, a forward primer was designed from the intergenic region upstream of CYP5122A1 gene that has not been included in the replacement construct (Primer F2 5′-GTTGCGACTTACCTTCTACGTGTG-3′; Fig. 4A). Two reverse primers were designed, one for each allele of the half knock out. The first reverse primer corresponded to sequences in the antibiotic resistance marker neomycin (Neo Internal Primer, 5′-CAAGGTGAGATGACAGGAGATC-3′; Fig. 4A) or the hygromycin resistance marker (Hyg Internal Primer, 5′-CAAGCAC TTCCGGAATCGGGC-3′) used to replace the CYP5122A1 gene and would identify the allele that has undergone homologous recombination. To identify the other allele, we designed a reverse primer (Primer P2 5′-CCTTCTTCCACTGCTCATCC-3′; Fig. 4A) in the region of CYP5122A1 gene that has been replaced on the corresponding allele and hence would only amplify fragments from the intact allele. We therefore expected amplification with primer pair F2/P2 only (Fig. 4C, lane 1) in the WT while the HKO parasites would give positive PCR reactivity with both F2/P2 as well as F2/Neo Int (Fig. 4C, lanes 3 and 4) or F2/Hyg Int (Fig. S4A (ii)).

To check the functional output of knockout of one allele, cell extracts from WT and HKO parasites were probed with anti-CYP5122A1 antibody to detect the extent of CYP5122A1 expression. Significantly lower amount of CYP5122A1 was expressed in the HKOs where replacement was made with the neomycin cassette (Fig. 4D, lane 1, Fig. 4E) and the hygromycin cassette (Fig. S4B, lane 2; S4C) as compared to WTs in respective blots. This knockdown was specific for CYP5122A1 because HKO parasites did not show any down-regulation of CYP710C1, another CYP450 gene of *Leishmania donovani* that was used as a CYP450 control (Fig. S4D). Protein expression was further confirmed by comparative fluorescence microscopy where staining intensity for CYP5122A1 was lower in HKO cells (Fig. 4F, c–d) as compared to WTs (Fig. 4F, a–b). To rule out the possibility of apparent higher staining due to larger size of the cell, mean fluorescence intensity per unit area per cell was measured microscopically. Fig. 4G shows the mean fluorescence intensity per unit area of the stained cells where the amount of CYP5122A1 staining in the HKOs/unit area was significantly less as compared to the WTs.

**Figure 5:**
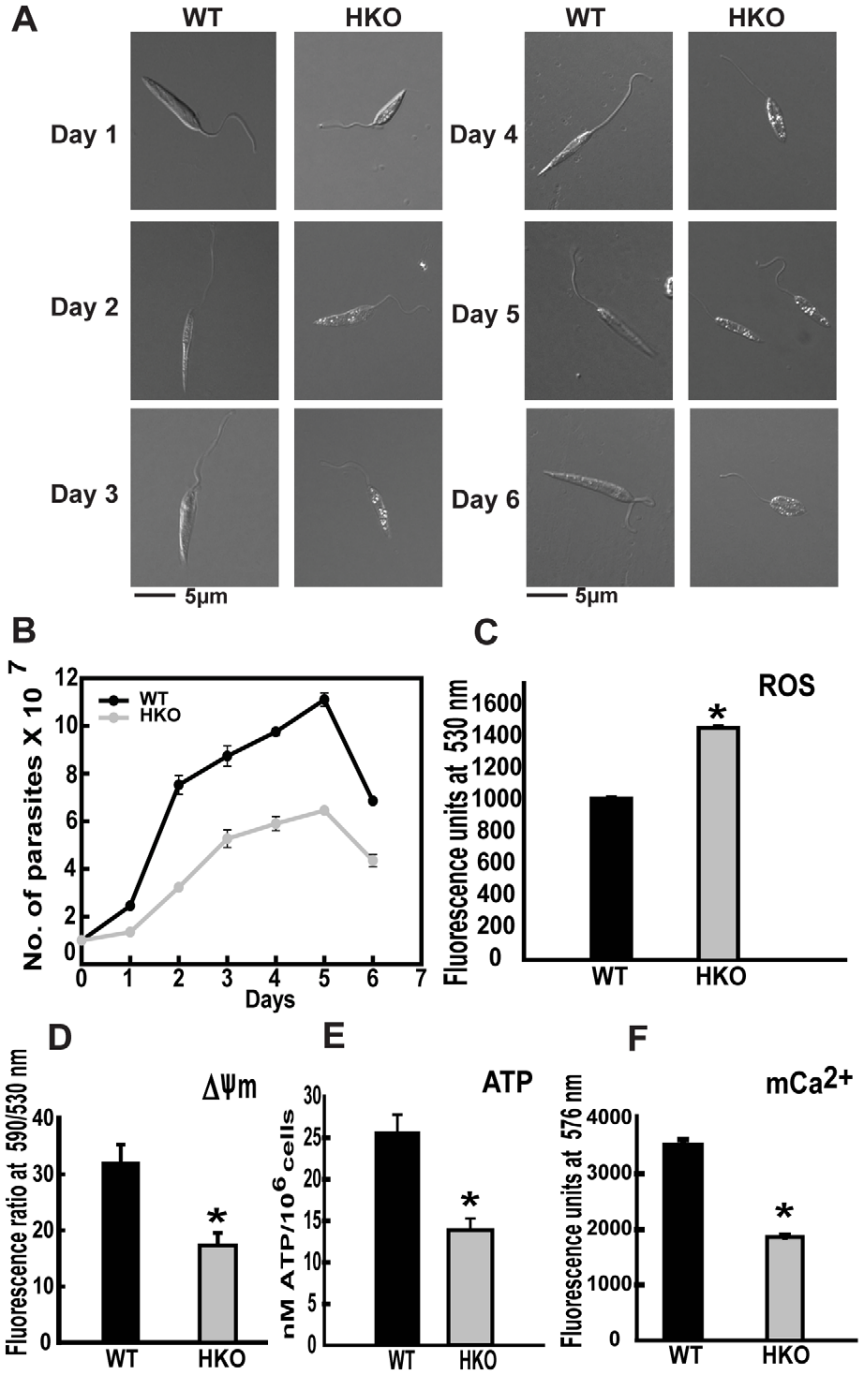
Morphology and biochemistry of HKO parasites. **A:** Photomicrographs of *L. donovani* WT and CYP5122A1 HKO cells during *in vitro* growth cycle. Scale 5 µm. **B:** Growth pattern of WT and HKO parasites *in vitro* over a period of 6 days in culture. Mean ± SE, n = 3. **C:** Comparison of average constitutive levels of intracellular ROS in WT and HKO parasites. Mean ± SE, n = 3, * P≤0.05. **D:** ΔΨm measured by the potentiometric probe JC-1. Mean ± SE, n = 3; * P≤0.05. **E:** Bar graph showing the basal level of ATP produced by the WT and the HKO cells. Mean ± SE, n = 3; *P≤0.05. **F:** Bar graph shows mitochondrial Ca2^+^ levels. Mean ± SE, n = 3; * P≤0.05.

To assess if the reduced expression of CYP5122A1 compromised cellular physiology, various parameters were checked in the HKO parasites. Morphologically, the HKOs were smaller in size than their WT counterparts at all stages of the *in vitro* life cycle (Fig. 5A). The division rate of the HKO cells was substantially slower than the WT cells showing a slower growth curve (Fig. 5B and Fig. S4E). HKO parasites in general were less motile unlike the highly motile WTs. Constitutive ROS level was higher in the HKOs as compared to WTs (Fig. 5C) when measured on various days of cycle. Impaired mitochondrial function in the HKOs was evident from lower mitochondrial membrane potential (ΔΨ_m_) and lower ATP levels as compared to WTs (Fig. 5D and E). Mitochondrial Ca^2+^, a known inducer of the generation of ΔΨ_m_ as well as the utilization of the ATP [22] was also lower in the HKO parasites (Fig. 5F). As evident from the above data, reduced expression of CYP5122A1 in the promastigotes compromised growth and mitochondrial function.

### Knockdown of CYP5122A1 compromises the parasites ability to infect both *in vitro* and *in vivo*

**Figure 6:**
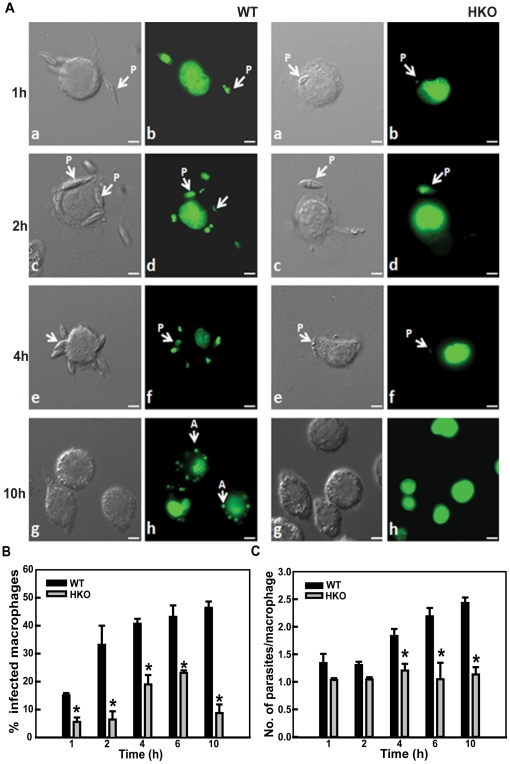
*In vitro* infective abilities of WT and HKOs. A. Photomicrographs of macrophage infected with WT and HKO parasites for different time points. b: 1 h; d: 2 h; f: 4 h; h: 10 h; a, c, e, g: nomarski of b, d, f, h respectively. Scale, 5 µm. **P:** promastigote, **A:** amastigote. **B.** Bar graph showing percentages of macrophages infected with WT and HKO parasites. Mean ± SE, n = 3, * P≤0.05. **C.** Number of parasites present in infected macrophages over a period of time. Mean ± SE, n = 3, * P≤0.05.

**Figure 7:**
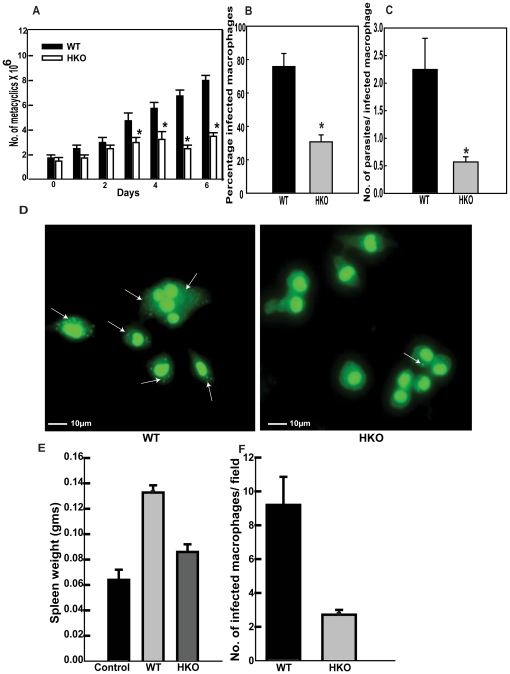
HKOs are impaired in metacyclogenesis and their ability to infect *in vivo*. **A.** Number of WT and HKO metacyclics during *in vitro* life cycle represented by bar graph. Mean ± SE, n = 3, * P≤0.05. **B:** Bar graph showing percentages of macrophages infected with WT and HKO metacyclics at an MOI of 1:10. Mean ± SE, n = 3, * P≤0.05. **C:** Number of parasites present in infected macrophages when infection was carried out with equal number of WT and HKO metacyclics. Mean ± SE, n = 3, * P≤0.05. **D:** Photomicrographs of macrophage infected with metacyclics (arrows) isolated from WT and HKO parasite cultures. Scale, 10 µm. **E:** Bar graph shows spleen weights of hamsters infected for 8 weeks. Control: uninfected hamsters; WT: hamsters infected with WT parasites, HKO: hamsters infected with HKO parasites. Mean ± SE ,n = 3, * P≤0.05. **F.** Infected macrophage numbers represented as the number of infectious foci per field in splenic smears from hamsters infected with WT (WT) and HKO parasites (HKO). Data represents mean ± SE, n = 5; in which 10 different fields were analysed. * P≤0.05.

Next, we sought to compare the infective abilities of the WT and the HKO parasites to assess if lesser CYP5122A1 expression would impair the parasites' ability to infect. Parasites containing the pBSK+ Neo cassette as replacement of one allele of CYP5122A1 showed reduced infecting abilities as compared to WT cells in terms of attachment at 2, 4 and engulfment at 6 h (Fig. 6A–C). Similarly, parasites containing the pBSK+Hyg cassette also showed a reduced ability to infect (Fig. S4G; % macrophages infected, control, 74±6; hygromycin HKO, 22±3, n = 3, *P≤0.05). The engulfed HKO parasites were cleared within 10 h of infection as suggested by the reduction in the number of macrophages containing internal parasites (Fig. 6B). This could be due to clearance by the macrophage generated ROS where HKO parasites would be more susceptible to such stress. This is further supported by the increased death of HKO promastigotes *in vitro* as compared to WTs when exposed to H_2_O_2_ (Fig. S5A). To assess the number of metacyclics in the inoculum of the HKO parasites to see if low number of metacyclics reduced infective abilities, peanut agglutinin based separation was used to isolate the infective forms. Lower numbers of metacyclics were recovered from the HKO cultures as compared to WT cultures (Fig. 7A) suggesting lesser differentiation if one allele of CYP5122A1 was removed. When the total number of metacyclics in the HKO inoculum was raised by increasing multiplicity of infection, there was a moderate increase in the number of macrophages infected but the infection was comparatively lower than infection achieved with the WT parasites (Fig. S5B). To investigate if the difference in infection rate was only due to the lesser number of metacyclics in the HKO cultures, macrophages were infected with equal numbers of metacyclics alone, isolated from both the WT and the HKO cultures. The infection pattern observed was similar to that obtained with total cellular cultures (Fig. 7B–D). This clearly showed that the HKO parasites cultures produced fewer metacyclics that were comparatively less efficient to infect as compared to metacyclics isolated from WT cultures.

Subsequently, to confirm the *in vitro* infection data *in vivo*, hamsters were used as a model system. Eight weeks after parasite administration to adult hamsters, significant splenomegaly was observed in animals infected with WT parasites as compared to the spleens in hamsters infected with HKO parasites as reflected in the comparative spleen weight (Fig. 7E). In consonance with the above observations, microscopic counts of splenic smears from HKO infected hamsters showed lesser number of macrophages containing parasites whereas splenic smears from WT parasite infected hamsters showed a large number of macrophages loaded with parasites (Fig. 7F). This data suggested that in the absence of full constitutive expression of CYP5122A1, the parasites demonstrated a compromised ability to infect both *in vitro* and *in vivo*.

### Knockdown of CYP5122A1 alters the parasites ability to withstand drug pressure

**Figure 8:**
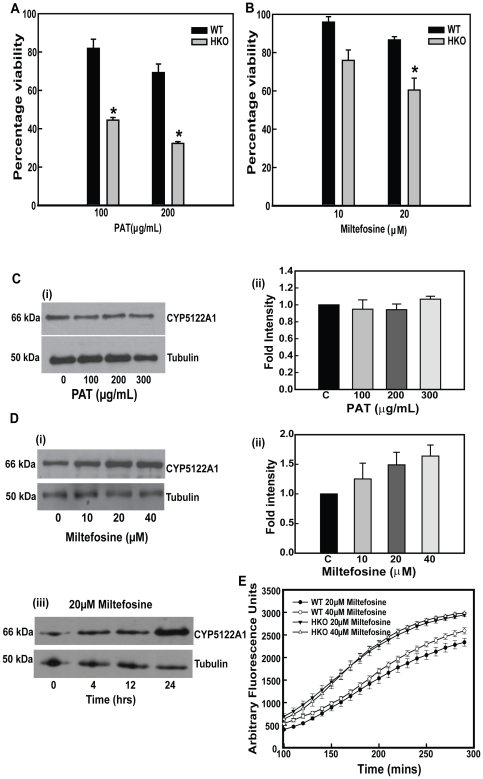
Effect of drug exposure. **A:** Bar graph shows percentage viability of WT and HKO parasites in response to treatment with PAT. Mean ± SE, n = 4; * P≤0.05. **B:** Bar graph shows percentage viability of WT and HKO parasites in response to treatment with miltefosine. Mean ± SE, n = 4; * P≤0.05. **C.** Western blot analysis (i) and its quantitation (ii) of total cell lysate from *Leishmania donovani* promastigotes post treatment with different doses of PAT (100 µg/mL, 200 µg/mL and 300 µg/mL) for 24 h probed with anti-CYP5122A1 antibody. **D.** (i) Western blot analysis of *Leishmania* total cell lysate post treatment with different concentrations of miltefosine for 24 h, (ii) quantitation of immunoblots in (i); (iii) western blot analysis of *L. donovani* whole cell lysate upon treatment with 20 µM miltefosine for different time periods. Mean ± SE, n = 4. **E.** Levels of intracellular ROS in response to treatment with miltefosine measured using the fluorogenic probe CMH_2_DCFDA. Mean ± SE, n = 3.

In the absence of vaccines, the treatment of visceral leishmaniasis has centred around several drugs like sodium stibogluconate (pentavalent antimony), amphotericin B and more recently, miltefosine [23]. The HKO parasites showed increased susceptibility to potassium antimony tartrate (PAT) the active form of pentavalent antimony and miltefosine (Fig. 8A–B, Fig. S4F) suggesting a role of CYP5122A1 in drug response. Therefore, based on the aforementioned results, WT parasites were checked for cellular level of CYP5122A1 after drug treatment. PAT was unable to induce any change in CYP5122A1 levels (Fig. 8C, (i) and (ii) whereas miltefosine was able to induce an up-regulation of CYP5122A1 at doses of 10–40 µM (Fig. 8D, i and ii) with maximal induction at 24 h with 20 µM of miltefosine (Fig. 8D, iii). Multiple drugs act via generation of ROS [24]–[25]. ROS levels measured after miltefosine exposure showed increased ROS in both the WT and the HKOs, generation in the HKOs being higher (Fig. 8E). Scavenging this ROS with the antioxidant N-acetyl cysteine (NAC, 20 mM), resulted in a substantial reduction of cell death in the HKOs (cell death %, control, 2±0.3; HKO, 35±4.0; HKO+NAC, 15±1.0, n = 3) suggesting that ROS generation was linked to drug efficacy. Therefore, the above observations strongly imply that a normal complement of CYP5122A1 is required by the *Leishmania* parasite for defense against drugs.

### Complementation of CYP5122A1 rescues phenotype

HKO parasites were complemented with WT copy of CYP5122A1 through episomal expression with the pXG-GFP+2 vector (expressing the CYP5122A1 gene). Fig S6A shows the presence of mRNA for CYP5122A1 in the transfected cells and the presence of the fusion protein was demonstrated by western blot and flow cytometry (Fig. S6B, C). The complementation resulted in rescue of morphology from shorter HKO cells to elongated forms resembling WTs (Fig. S6D). When drug susceptibility of the HKO parasites was compared to the parasites with CYP5122A1 complementation, the complemented parasites were comparatively more resistant to drugs as compared to the HKOs but not as efficient as WTs to resist drug induced death (Fig. S6E). This fusion protein also localized in the ER of the parasites as seen by a co-localization with ER Tracker blue white DPX stain (Fig. S6F).

### Changes in ergosterol occur with alterations in CYP5122A1 levels

**Figure 9:**
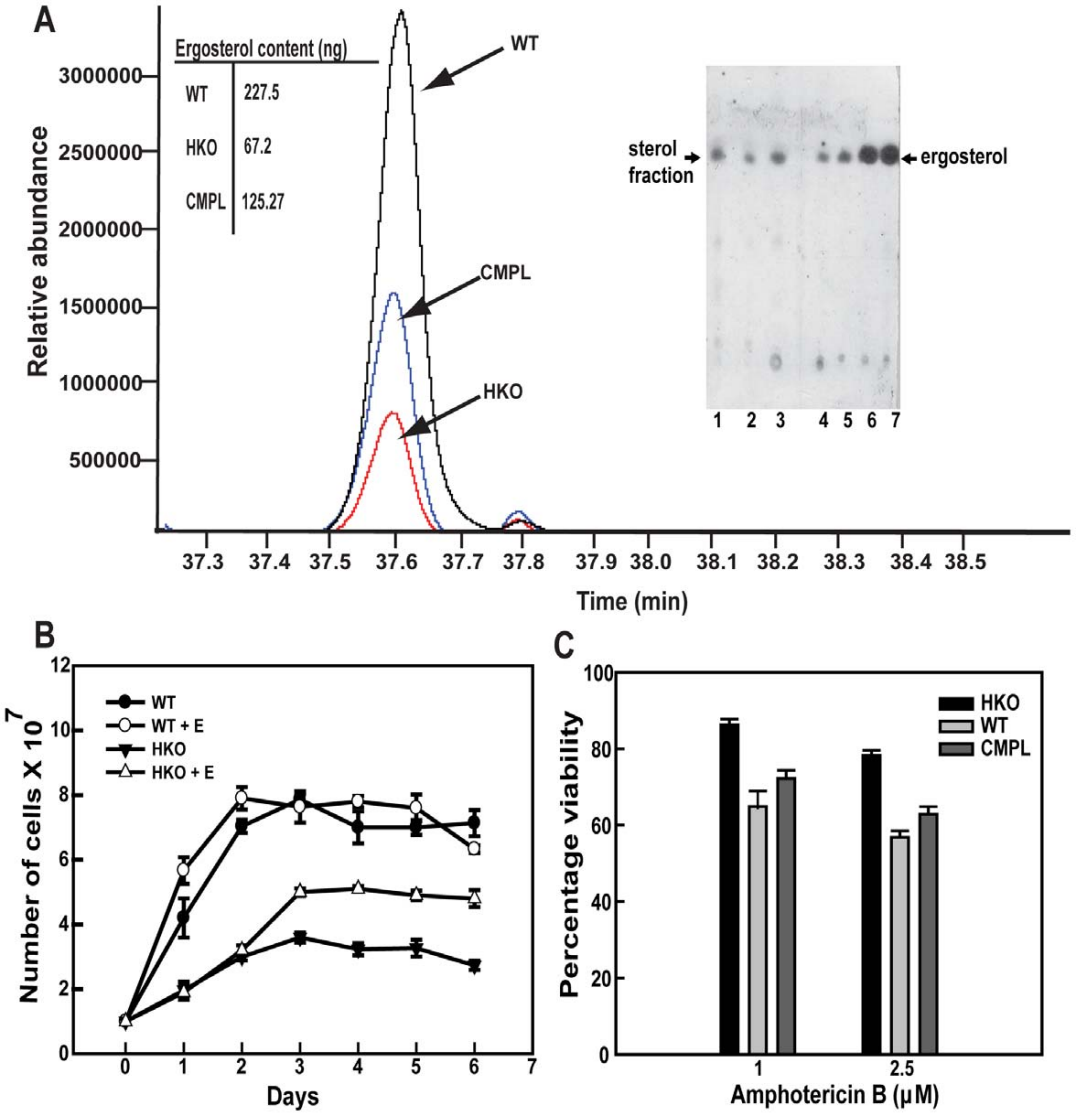
Synthesis of ergosterol in HKO and WT parasites. **A.** Chromatogram of ergosterol profile from sterol extracts of WT, HKO and CMPL strains subjected to GC-MS analysis. **Insets.** Table showing the approximate ergosterol content as calculated using a standard curve. TLC analysis of ergosterol from WT (Lane 1), HKO (Lane 2) and complemented parasites (Lane 3). Rf value of ergosterol: 0.588. Standard ergosterol dilutions (Lane 4: 0.05 µg, Lane 5: 0.1 µg, Lane 6: 0.5 µg, Lane 7: 1 µg). **B.** Comparative growth pattern of WT and HKO parasites *in vitro* over a period of 6 days in culture, in the presence and absence of ergosterol. Mean ± SE, n = 3. **C.** Bar graph shows percentage viability of WT, HKO and CMPL parasites in response to treatment with amphotericin B for 6 h. Mean ± SE, n = 3.

Since several CYP450s are functionally related to sterol biosynthesis [26], the HKOs carrying one allele of CYP5122A1 were checked for ergosterol content. HKO parasites expressed 3.5 times lesser ergosterol than the WTs as evident from GC-MS based analysis of organic solvent extracts of the cells (Fig. 9A). Although it is not known if the *Leishmania* parasite can incorporate ergosterol *in vitro*, a related kinetoplastid parasite, *Trypanosoma brucei* takes up sterol precursors in the host [27]. Ergosterol supplemented *in vitro* during *Leishmania* culture showed an uptake of ergosterol, and a higher HKO parasite growth rate in contrast to those cultured without ergosterol supplementation (Fig. 9B). Presence of a higher level of ergosterol in CYP5122A1 complemented HKO parasites indicated CYP5122A1 involvement in maintaining ergosterol levels, although the extent of recovery was not equivalent to the WTs (Fig. 9A). The alteration of sterol levels could have important implication in drug response because ergosterol is required for action of some drugs. For example, amphotericin B, a polyene antifungal drug also used for the treatment of leishmaniasis [1] associates with ergosterol for its action [28], arguably therefore, cytotoxic effect of amphotericin B would be lesser in the HKO parasites. There was a distinct lowering of amphotericin B cytotoxicity in CYP5122A1 HKOs in comparison to the WTs (Fig. 9C) as evident from the percentage viabilities. Therefore, this data clearly indicated that deficiency in CYP5122A1 resulted in lowering of ergosterol levels consequently making them less susceptible to amphotericin B.

## Discussion

Cytochrome P450s are an important group of proteins involved in the synthesis of physiologically active compounds, drug metabolism and bioconversion of xenobiotics. Although the parasite genome database annotations identify 3 distinct CYP450s in *Leishmania donovani*, no data is available on the possible functions of these proteins in this medically important parasite. Both from the point of view of identifying drug targets and understanding the physiology of the *Leishmania* parasite, CYP450s need to be investigated. In this study, we report the involvement of one of the CYP450s, the CYP5122A1 in the survival and function of the *Leishmania donovani* parasite.

The sequence of the cloned CYP450 from *L. donovani* obtained using primers designed from *L. major* showed the presence of signature motifs of CYP450 proteins. Designated CYP5122A1 by the CYP450 Nomenclature Committee, the sequence of the protein showed close identity within the subgroup of *Leishmania* but shared ∼50% identity with the *Trypanosoma* sequences. For functional studies, the free-swimming *L. donovani* promastigotes were selected as an ideal model system to analyse the function of CYP5122A1 because promastigotes differentiate into infective metacyclics that eventually infect mammalian macrophages. No significant alterations in the CYP5122A1 levels during *in vitro* life cycle suggested constitutive levels to be sufficient for growth and metacyclogenesis. However, the higher expression of the protein in stationary phase metacyclics as compared to the log phase ones implied that accumulation of this protein was coincident with increase in infective abilities of the parasites. Interestingly, the intracellular amastigotes also expressed a higher quantity of the protein than promastigotes. The requirement of CYP5122A1 for normal growth of the parasite was substantiated by impaired differentiation and growth of the HKO parasites where one allele of CYP5122A1 was replaced by antibiotic resistance resulting in expression of half the amount of the protein as is present in the WTs. When HKO parasites were transfected to episomally express CYP5122A1 to compensate the reduced expression in them, improved growth as compared to the HKOs further validated the requirement of CYP5122A1 for development of the parasite. The sharp decline in ergosterol levels in the HKO parasites coupled with their inability to tolerate oxidative stress indicated that the lowered ergosterol could be a reason for lesser parasite growth and lack of resistance to drug-induced stress. While the improved growth rate upon supplementation of ergosterol could be looked upon as an indication of a positive role of the sterol, the HKO parasites episomally expressing CYP5122A1 showing increased ergosterol content and improved growth rate further confirmed the importance of ergosterol. In another kinetoplastid parasite, the *Trypanosoma* spp. alterations in ergosterol levels through enzymatic inhibition induce reduction in cell growth [29]–[30]. Although *Leishmania* parasites can survive with lower sterol levels, normal supplement of sterol is required for optimal parasite function [17] which is reinforced by this study. Since sterol biosynthesis inhibitors are projected as attractive drug targets [16], [31], lower ergosterol in the HKOs suggest a link of CYP5122A1 to sterol biosynthesis which makes a strong case for CYP5122A1 to be investigated further for evaluation as a possible drug target.

Efficient ability to bind to the macrophages and invade the cells determines the extent of infection and finally, disease pathogenesis. Reduced infective ability of the HKO parasite when compared to WT parasite could be due to several reasons. Firstly, reduced infectivity could be related to lower number of infective forms present in the HKO parasite inoculum, secondly, defects in host-parasite membrane interaction or thirdly, a rapid parasite clearance by host generated ROS. The lower recovery of metacyclics from the HKO cultures as compared to the WT cultures indicated that formation of fewer metacyclics could be a reason for lower infectivity. Since metacyclics from HKOs were less efficient for infection as compared to the WT metacyclics, the quality of their membrane could also be a reason for impaired ability to infect. Therefore, it is likely that a combination of factors like lesser number of infective forms in a given inoculum and defective membrane fusion events were responsible for lower infection rate by the HKO parasites. Altered lipid composition of membranes is known to affect membrane binding events in general like in yeast where ergosterols play an important role in plasma membrane fusion [32]–[33]. HKO parasites expressing low ergosterol were cleared faster by the host possibly because of compromised resistance to oxidative stress as sterols are proposed to be crafted by natural selection as an early defense mechanism to ROS [15]. The notion that parasites with low ergosterol are vulnerable to oxidative stress is further supported by increased susceptibility of these parasites to H_2_O_2_ induced stress *in vitro*. Since the extent of sterol oxidation is defined by the sterol content itself, the low ergosterol cells would be prone to increased ROS induced damage [34].

As low CYP5122A1 levels in the HKO parasites increased oxidative stress susceptibility, it was of interest to investigate how the WT parasites expressing normal CYP5122A1 levels handled drug exposure. The WT parasites resisted lower doses of miltefosine more efficiently because they could up-regulate CYP5122A1 in response to the drug. Altered lipid content by the action of miltefosine [35]–[36] combined with increased miltefosine-generated ROS increased the susceptibility of the HKO parasites to the drug. It was obvious that ROS played a major role in miltefosine induced cell death because presence of an antioxidant during miltefosine exposure protected the cells. In contrast, PAT treatment could not induce CYP5122A1 increase and caused higher death. Although the difference in response could be due to the dissimilar mode of action, these observations lend credence to the suggestion that CYP5122A1 levels are critical for combating drug effects. Arguably, drugs using cellular sterols to exert their action should show impaired effects in the HKO cells with lower ergosterol. Consistent with this view, HKO parasites were less susceptible to amphotericin B, an anti-leishmanial drug that requires association with membrane ergosterol for action [9], [18]. This provides an important possibility that CYP5122A1 deficiency could be linked to resistance to amphotericin B as changes in sterol profile is known to accord amphotericin B resistance [9], [18].

The mitochondria primarily sequesters Ca^2+^ to stimulate and control the rate of oxidative phosphorylation to induce mitochondrial permeability transition, regulate cytosolic Ca^2+^ pulses and drive apoptotic cell death [22]. The partially functional mitochondria, with the lower ΔΨ_m_, low ATP production, low Ca^2+^ and higher ROS generation in HKO parasites possibly reflected consequences of lower ergosterol content as well because ergosterol is found in the membranes of intracellular organelles including *Leishmania* mitochondria [37].

The close similarity in CYP5122A1 sequences between the old and the new world *Leishmania* spp. with the conserved heme binding domain suggest commonality of function with its orthologues in the *Leishmania* and *Trypanosoma* species. Therefore, how can this data be integrated in a model of *Leishmania* survival? The information obtained clearly establishes that interference with CYP5122A1 levels have deleterious consequences for the parasite in terms of compromised organellar function, increased susceptibility to drugs and impairment of the ability to infect, firmly establishing CYP5122A1 as an essential protein for *Leishmania* survival. Therefore, it would be of interest to check the status of this protein in *Leishmania* field isolates from endemic regions to relate if alterations of this protein is linked to drug resistance. The data presented here strongly suggests that CYP5122A1 could be a possible drug target as it is functionally important for parasite survival and does not share identity with host CYP450s making it amenable to interception. Moreover, this protein could play an important role in resistance to drugs that use sterols to exert their effects, for which the levels of this protein in drug resistant isolates from endemic areas needs to be investigated.

## Materials and Methods

### Reagents

ERTracker™ Blue-White DPX, MitoTracker® Red, Rhod – 2AM, JC-1 (5,5′,6,6′-tetrachloro-1,1′,3,3′-tetraethylbenzimidazolylcarbocyanineiodide), CM-H_2_DCFDA, SYTO 11 Green, ATP determination kit and pluronic acid were obtained from Molecular Probes (Eugene, OR). pGEM-TEasy vector system was procured from Promega (Madison, WI). Trizol, First strand c-DNA synthesis kit and Hi-fidelity *Taq* polymerase were from Invitrogen (Carlsbad, CA). Restriction enzymes, dNTPS, Quick ligase, T4 DNA ligase, and *Taq* polymerase were from New England BioLabs (Beverley, MA). Miltefosine was purchased from Cayman Chemicals (Ann Arbor, MI). Potassium antimony tartrate (PAT), Dulbecco's Modified Eagles Medium (DMEM), ergosterol, peanut agglutinin, MTT (3-(4,5-Dimethylthiazol-2-yl)-2,5-diphenyltetrazolium bromide), triton-X-114, amphotericin B, BSTFA (N,O-bis (trimethylsilyl) trifluoroacetamide), TMCS (trimethylchlorosilane), foetal bovine serum and genomic DNA isolation kit were obtained from Sigma Aldrich. Western blotting reagents and enhanced chemiluminescence kit were from Amersham Life Sciences (Piscataway, NJ). CB-X™ Protein assay kit was obtained from G-Biosciences (Maryland Heights, MO). Silica G plates were from Analtech Inc. (Newark, DE). Peptides were commercially synthesized by USV Peptides Ltd. (Bangalore, India). All other chemicals, unless otherwise specified, were from Sigma Aldrich.

### Antibodies

Anti-CYP5122A1 antibody was raised in rabbits using a peptide specific for the CYP5122A1 protein (Gly-Pro-Arg-Gly-Val-Pro-Ser-Val-Asn-Asp-Val-Arg-Asp-Leu). This stretch was selected for raising antibodies for its high antigenicity, hydrophobicity, the presence of coils in the secondary structure and sequence specificity for CYP5122A1 distinct from the other CYP450s annotated in the *Leishmania* spp. database. Similarly antibody was also raised against another CYP450 of *L. donovani*, CYP710C1 using the peptide (Cys-Asp-Glu-Lys-Thr-Val-Val-Pro-Lys-Gly-Met-Ile). Rabbit anti-tubulin-α antibody was obtained from Neomarker (Fremont, CA). Anti-BIP antibody was a kind gift from Dr. J. D. Bangs, University of Wisconsin (Madison, WI). Secondary antibodies conjugated to Alexa Flour 488 and 594 and horse radish peroxidase were obtained from Invitrogen (Carlsbad, CA) and Jackson Immunoresearch Laboratories (West Grove, PA) respectively. Anti-GAPDH was from Santa-Cruz Biotechnology (Santa Cruz, CA).

### Animals

Syrian hamsters (*Mesocricetus auratus*), 3–6 weeks old, were used for *in vivo* infection.

### Ethics statement

All procedures in this study were carried out as per the guidelines laid down by the “Institutional Animal Ethics Committee” of the National Institute of Immunology, New Delhi. The protocol for this specific study was approved by the same committee via permit no. IAEC/168/07. This study was also approved by the “Institutional Biosafety Committee” via permit no. IBSC110#07.

### Cells, treatments, extraction and MTT assay

*Leishmania donovani* promastigotes, amastigotes and J774A.1 murine macrophages (ATCC No. TIB-67) were cultured as described earlier [6], [7], [38]. Metacyclics were isolated by incubating 10^7^ cells in 100 µg/mL peanut agglutinin (PNA) and cells non-adhered to PNA (metacyclics) were separated by centrifugation [21]. To study effects of drugs, parasites in the logarithmic phase were treated with different doses of miltefosine (10 µM to 40 µM), potassium antimony tartrate (100–300 µg/mL) and amphotericin B (1–2.5 µg/mL). For extraction of membrane proteins, cells were resuspended in 0.5% Triton X-114, homogenized and incubated on ice for 90 mins. The insoluble material was removed by centrifugation and the supernatant was incubated at 37°C for one hour following which the aqueous and detergent phases were separated. Ergosterol was added to culture medium at a final concentration of 3 µg/mL. To estimate cytotoxicity, MTT assay was carried out as described previously [38].

### Cell lysates, SDS-PAGE and western blotting

Cell lysis was performed in a lysis buffer (0.125 M Tris HCl, (pH 6.8) 4% SDS, 20% v/v glycerol, 10% β-mercaptoethanol) and protein content was quantitated using CB-X™ Protein Assay Kit. SDS PAGE, western blots and densitometry were carried out as described previously [6], [38]. For western blots, antibodies were used at various dilutions; anti-CYP5122A1 antibody (1∶20,000), anti-tubulin-α antibody (1∶10,000), secondary antibodies (1∶20,000 dilutions). Reactivity was visualized by enhanced chemiluminescence.

### Cloning, sequencing and analysis

Overlapping sets of primers for the CYP450 protein genes were designed from sequence available with the *Leishmania major* genome database (LmjF27.0090, Gene DB hosted by the Wellcome Trust Sanger Institute, Hinxton, UK). Using *L. donovani* genomic DNA as template, an insert was amplified with relevant primers and cloned into pGEM-TEasy vector. Sequencing was carried out by the di-deoxy method [39], at sequencing facility of Delhi University (New Delhi, India). The sequence was submitted to the CYP450 Nomenclature Committee [19] (http://drnelson.uthsc.edu/cytochromep450.html) for assignment. Alignment of the *L. donovani* sequence with other CYP450s was performed using ClustalW hosted at the European Bioinformatics Institute [40]. The protein sequence derived was subjected to secondary structure prediction program PSIPRED hosted at the Bloomsburry Center for Bioinformatics [41]. The derived protein sequence of CYP5122A1 was also submitted to the SWISS-MODEL homology modelling server hosted at the Swiss Institute of Bioinformatics [42]. The model generated was viewed in Pymol molecular graphic software (educational use). The stereochemical qualities of this 3D model were checked using Ramachandran plot [43] generated by PROCHECK (EMBL-EBI, UK) (http://www.ebi.ac.uk/thornton-srv/software/ PROCHECK/). WHATCHEK (http://www.cmbi.kun.nl/gv/whatcheck/) server was used to assess the packing quality of the protein model [44].

For CYP5122A1 allelic replacement constructs, ORFs encoding neomycin and hygromycin resistance were inserted between 0.4 kb of 5′ and 0.37 kb of 3′ of CYP5122A1 homologous regions in the pBlueScript SK+ vector to generate the constructs pBSK+CYP5122A1Neo and pBSK+CYP5122A1Hyg respectively. Half knockouts (HKOs) were generated with these constructs through transfection of log phase promastigotes as described previously [38]. To generate double knockout parasites, second allelic replacement construct was transfected into the HKO parasites. Proper insertion of cassettes was confirmed by PCR to detect the presence of replacement constructs into the transformant genomic DNA. Primers were also designed using the intergenic sequence upstream of CYP5122A1 gene, the antibiotic resistance marker ORFs and the CYP5122A1 gene, such that amplification using these primers of expected molecular weight would only occur upon insertion at the correct locus. The leishmanial expression vector pXG-GFP +2 (a kind gift from Dr. S. Beverley) containing the CYP5122A1 gene was used for complementation of CYP5122A1 through episomal expression in the HKO parasites. Complementation was confirmed by GFP expression and detection of fusion m-RNA and GFP-fusion protein in the transformants.

### Immunocytochemistry and growth curve

Fixed (2% formaldehyde), digitonin or saponin (0.01%) permeabilized cells were stained using rabbit anti-CYP5122A1 antibody (1∶500) followed by labelling with Alexa Fluor 488 conjugated secondary antibody (1∶750). For co-localization, MitoTracker® Red (0.5 µM/10^6^ cells) was used to visualize mitochondria while ER-Tracker™ Blue-White DPX (1 µM/10^6^ cells), anti-BIP antibody (1∶1000) were used to identify the endoplasmic reticulum (ER). Anti-GAPDH antibody (1∶50) was used to identify glycosomes. Stained cells were analysed with a Nikon Eclipse TE2000E microscope (Nikon, Japan). FACS Calibur (Becton Dickinson, NJ) equipped with a 488 nm air cooled argon ion laser was used for flow cytometric analysis as described previously [45]. Mean fluorescence intensities per unit area of WT and HKO cells stained with anti-CYP5122A1 antibody were measured using the software ImagePro Plus (Media Cybernetics). For growth curve, equal number of cells (10^7^ or 5×10^6^) was plated and cell numbers analysed by haemocytometer based counting every 24 h.

### Measurement of ΔΨ_m_, Ca^2+^, ROS and ATP

Ca^2+^ and ΔΨ_m_ was measured using Rhod-2 AM and JC-1 as probe as described previously [6]. For measurement of ROS, the cells (10^7^) were loaded with the cell permeant probe CM-H_2_DCFDA (2 µg/mL) as described previously [6] and fluorescence measurements were taken at 507/530 nm using a FluoStar Optima fluorescence reader (BMG Labtechnologies Inc., Germany). ATP was measured in a reaction buffer containing 1 mM dithiothreitol, 0.5 mM luciferin and 12.5 µg/mL luciferase using a Fluostar Omega multi-label detection system as described previously [6].

### Infection

*In vitro Leishmania*-macrophage infection was carried out as described previously [7] at a parasite: macrophage ratio of 10∶1. For *in vivo* infection, Syrian golden hamsters were injected intra-cardially with 10^8^ parasites in stationary phase of growth. After 8 weeks, the animals were euthanized and spleen slices were used to prepare splenic smears. Smears were stained with Giemsa stain and scored for infected macrophages.

### Estimation of ergosterol

Sterols were isolated as described by Arthington-Skaggs et al. [46], with slight modifications. Briefly, cells were suspended in 25% alcoholic KOH and incubated at 85°C (1 h) followed by cooling to room temp and addition of sterile distilled water and n-heptane mixture. Standard ergosterol dilutions and sterol extraction samples from WT, HKO and complemented strain of parasites were spotted on a Silica Gel G plate, resolved using a binary solvent (hexane/ethyl acetate (75/25)) and visualized using Mo's stain (12.5 g of ammonium molybdate (VI) tetrahydrate, 5.0 g of ammonium cerium (IV) sulphate, 50 ml of concentrated sulfuric acid volume raised to 500 mL with water). For GC-MS, the sterol extracts were dried under nitrogen gas, resuspended in *n*-hexane and derivatized with BSTFA containing 1% TMCS at 70°C for 1 h. The derivative mixture was dried under nitrogen to remove excess BSTFA and subsequently re-dissolved in *n*-hexane. Agilent 7890A gas chromatography instrument coupled to an Agilent 5975C mass spectrometer and an Agilent ChemStation software (version G1701EA, Agilent Technologies, Palo Alto, CA) was used for GC-MS. For separation, a HP-5MS capillary column (30 m×0.25 mm i.d) was used with the column temperature was set at 100°C (held for 5 min for injection) programmed at 20°C min^−1^ to 200°C (held for 10 min, followed by 10°C min^−1^ to 230°C) and finally at 5°C min^−1^ to 320°C (5 min). Injection temperature was set at 260°C. High purity helium was used as carrier gas of 1.0 mL min^−1^ flow-rate. The spectrophotometers were operated in electron-impact (EI) mode, full scan of 40–550 amu or selected ion monitoring (SIM) was used, the ionization energy was 70 eV. Calibration curve was generated using *n*-hexane stock solutions of standard ergosterol dilutions (5- 500 ng/mL) in duplicates and plotting the peak area versus the concentration.

### Statistical analysis

Data were analysed by student's unpaired *t*-test. Values were considered significantly different at P≤0.05. All experiments were repeated 3–4 times. Data are expressed as the mean ± SE of several independent experiments.

## Supporting Information

Figure S1 **Phylogenetic analysis of CYP5122A1.** A phylogram generated from CYP450-like sequences analysed by ClustalW showing distances between CYP450-like proteins of various species. Scale represents 0.06 nucleotide substitutions per site.
(TIF)

Figure S2 **Predicted secondary structure.** Schematic representation of the predicted secondary structures in and around the active site generated using the PSIPRED server hosted at the Bloomsburry Centre for Bioinformatics (University College, London and Birkbeck college). PTG, Proton transfer groove; SC, stabilization core; HBL, Heme binding loop are highlighted in yellow. Active site cysteine indicated by arrow.
(TIF)

Figure S3 **Ramachandran plot of CYP5122A1.** Ramachandran plot generated for CYP5122A1 protein structure modelled using SWISS-MODEL shows majority of the residues to fall into the favourable region of the plot.
(TIF)

Figure S4 **Analysis of pBSK+CYP5122A1-Hyg HKO.** **A:** (**i**) Insertion of allelic replacement construct into the genomic DNA was confirmed by PCR for hygromycin and knockout-hygromycin construct using primers that span the 5′ homologous sequence (Forward primer) and the hygromycin gene (Reverse primer). Lane 1: fragment amplified for the hygromycin resistance gene; lane 2: 1 kb DNA ladder; lane 3: fragment amplified for the knock out hygromycin construct. (ii) Confirmation of insertion of the replacement construct at the correct locus was assessed by PCR. Lane 1: amplicons generated from HKO genomic DNA as template with primers F2/P2 indicating the presence of an intact CYP5122A1 allele (primer positions indicated in schematic in Fig. 4A); Lane 2: amplicons generated from HKO-Hyg genomic DNA as template with primers F2/HI indicating the presence of insertion of hygromycin resistance ORF at the correct locus. **B:** Western blot analysis of cell lysates from WT and HKO parasites with anti-CYP5122A1 antibody showing the decreased level of CYP5122A1protein in the HKOs. Loading was normalized with Tubulin–α (50 kDa). **C:** Bar graph representing averaged densitometric analysis of immunoblots represented in B, mean± SE, n = 3. **D:** Western blot analysis of cell lysates from WT and HKO parasites using anti-CYP710C1 antibody showing equal levels of the CYP710C in the WT and the HKO parasites. Loading was normalized with tubulin-α (50 kDa). **E:** Comparison of growth and survival of WT and HKO parasites *in vitro* over a period of 6 days in culture. Mean ± SE, n = 3. **F:** Bar graph showing percentage viability of WT and HKO parasites in response to treatment with PAT. Note the increased susceptibility of HKOs as compared with WT. Mean ± SE, n = 4; * P≤0.05. **G:** Photomicrographs of macrophage infected with WT and HKO parasites stained with Syto 11 Green fluorescent nucleic acid stain. **a**: nomarski image; **b**: Syto 11 Green stained image; **c**: merge of a and b. Scale, 5 µm.
(TIF)

Figure S5 **A:** Bar graph shows percentage viability of WT and HKO parasites in response to treatment with H_2_O_2_. Mean ± SE, n = 4; * P≤0.05. **B:** Photomicrographs of J774A.1 macrophages infected with WT and HKO *L. donovani* parasites at multiplicity of infection at 1∶25 and 1∶50, stained with Syto 11 Green. Scale, 20 µm.
(TIF)

Figure S6 **Effects of complementation of CYP5122A1 in HKOs.** **A:** Agarose gel (1.5%) showing expression of GFP-CYP5122A1 fusion mRNA (GFP Forward primer & CYP5122A1 internal primer) in 2 clonally selected strains of CYP5122A1 complemented parasites. Lane 1: negative control; lane 2 & 3: fusion product amplified from c-DNA prepared from complemented parasites; lane 4: 1 kb DNA Ladder; lane 5: Positive control amplified using pXG-GFP+2CYP5122A1 plasmid as the template. **B:** Western blot analysis of lysates of HKO and CMPL cells probed for the fusion protein show the presence of GFP-CYP5122A1 protein at expected molecular weight of approximately 96KDa in lysates of complemented parasites. **C:** Flow cytometric analysis of complemented parasites. **D.** Photomicrographs of HKO (a) and complemented (b) parasites. Scale, 5 µm. **E:** Bar graph showing percentage viability of WT, HKO and complemented parasites in response to treatment with miltefosine and PAT. Mean ± SE, n = 4; * P≤0.05. **F:** Photomicrographs of CMPL promastigotes stained with anti-CYP5122A1 antibody and ER-specific markers, ER Tracker Blue-White DPX. Scale, 5 µm.
(TIF)

Figure S7 **Subcellular localization of CYP5122A1 in metacyclic parasites** Photomicrographs of *Leishmania donovani* metacyclic parasites stained with anti-CYP5122A1 antibody and ER-specific marker, BIP. Scale represents 10 µm.
(TIF)
