# Supplementary figures and images for: CYP5122A1, a Novel Cytochrome P450 Is Essential for Survival of Leishmania donovani
Source: PLoS One. 2011 Sep 23;6(9):e25273. doi: 10.1371/journal.pone.0025273 (PMC3179497; doi:10.1371/journal.pone.0025273)

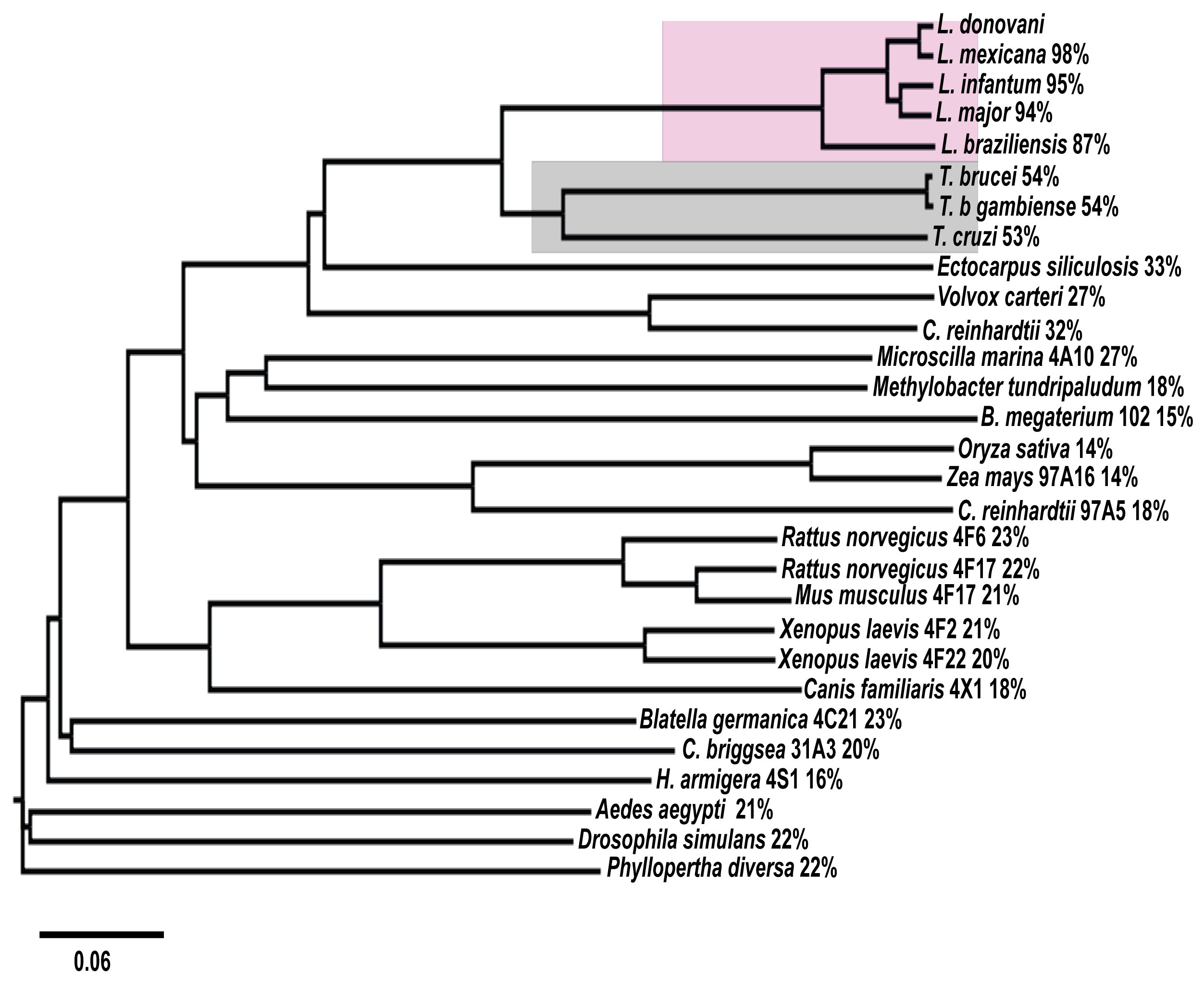

Supplement: Figure S1 — Phylogenetic analysis of CYP5122A1. A phylogram generated from CYP450-like sequences analysed by ClustalW showing distances between CYP450-like proteins of various species. Scale represents 0.06 nucleotide substitutions per site. (TIF) [file pone.0025273.s001.tif]

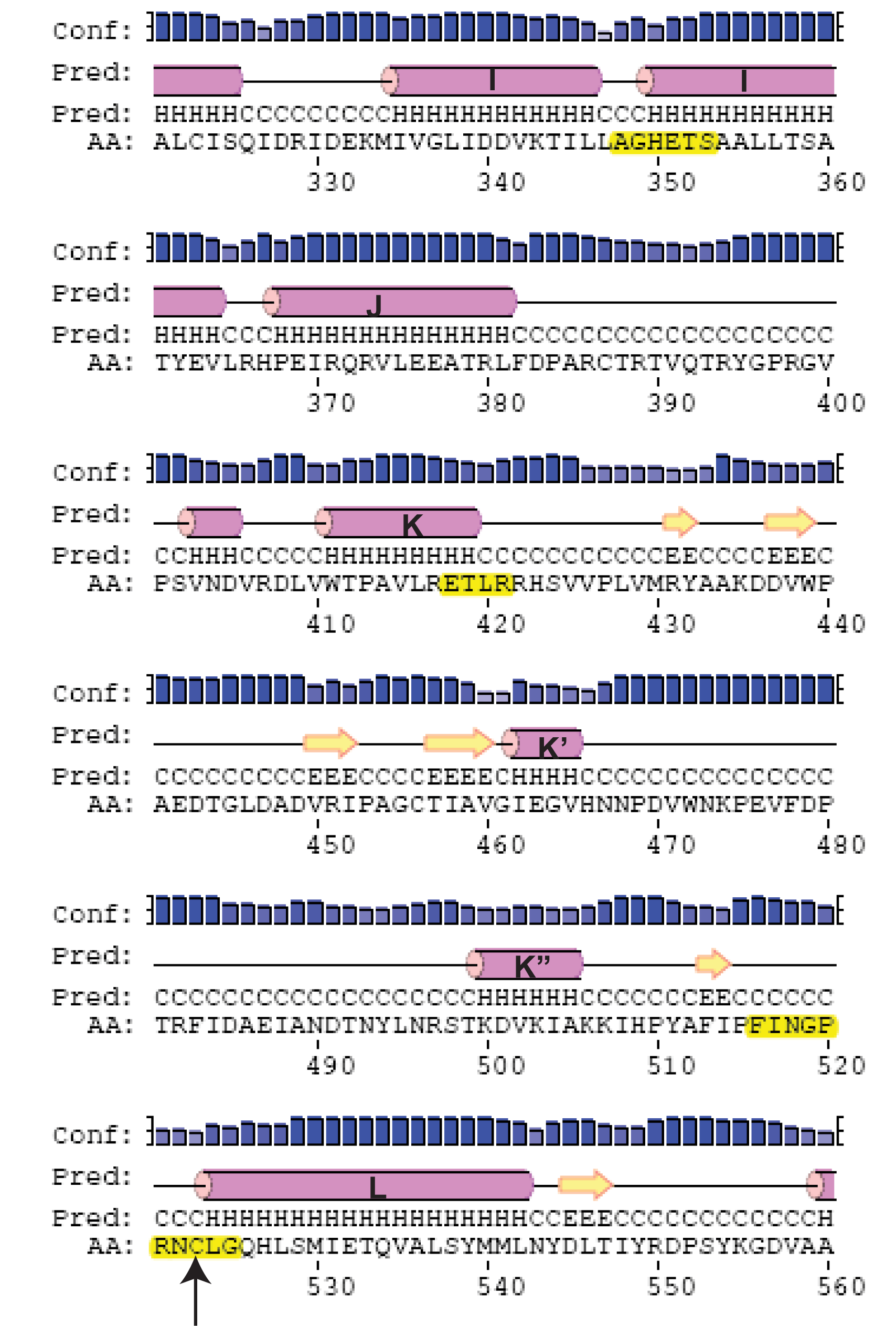

Supplement: Figure S2 — Predicted secondary structure. Schematic representation of the predicted secondary structures in and around the active site generated using the PSIPRED server hosted at the Bloomsburry Centre for Bioinformatics (University College, London and Birkbeck college). PTG, Proton transfer groove; SC, stabilization core; HBL, Heme binding loop are highlighted in yellow. Active site cysteine indicated by arrow. (TIF) [file pone.0025273.s002.tif]

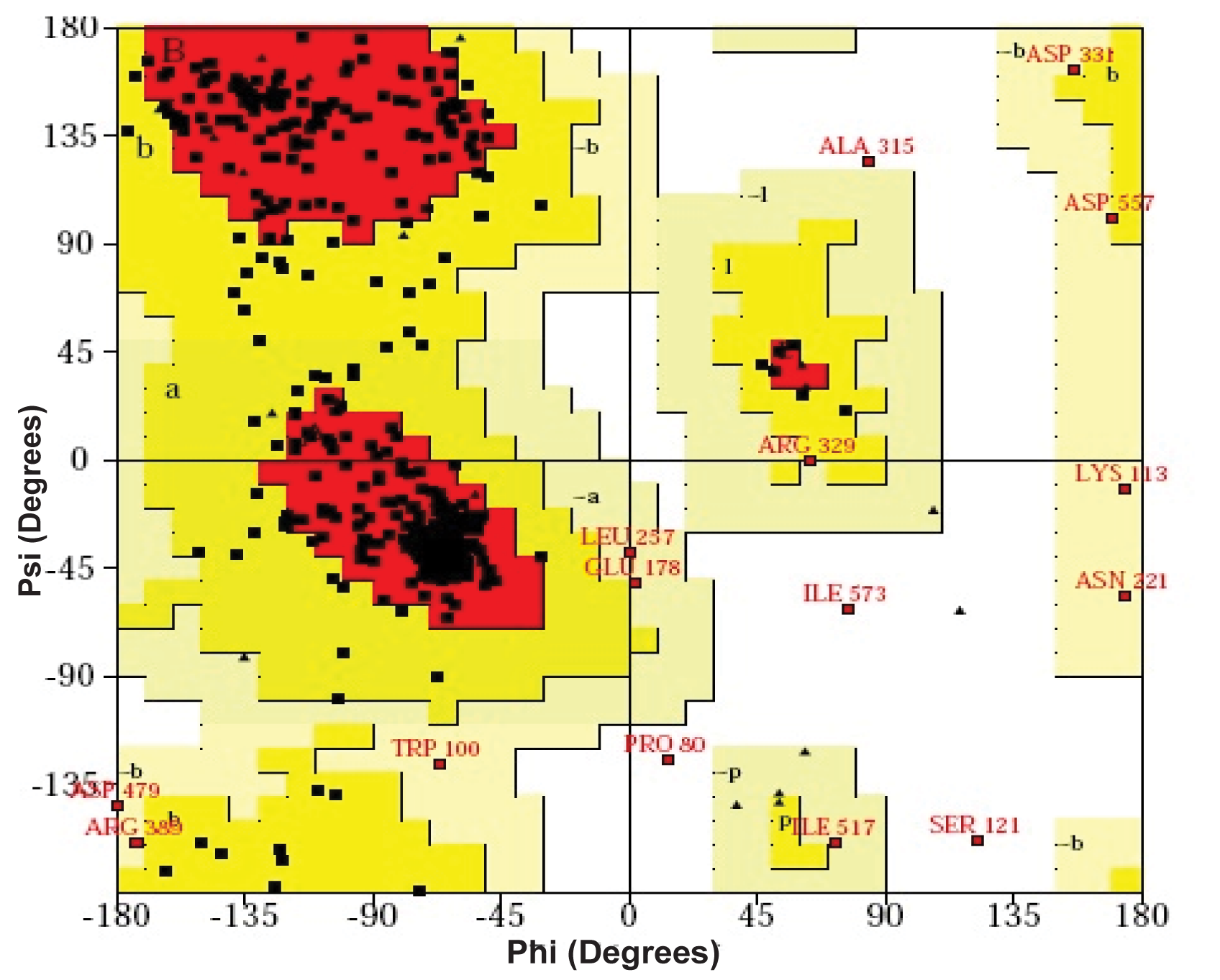

Supplement: Figure S3 — Ramachandran plot of CYP5122A1. Ramachandran plot generated for CYP5122A1 protein structure modelled using SWISS-MODEL shows majority of the residues to fall into the favourable region of the plot. (TIF) [file pone.0025273.s003.tif]

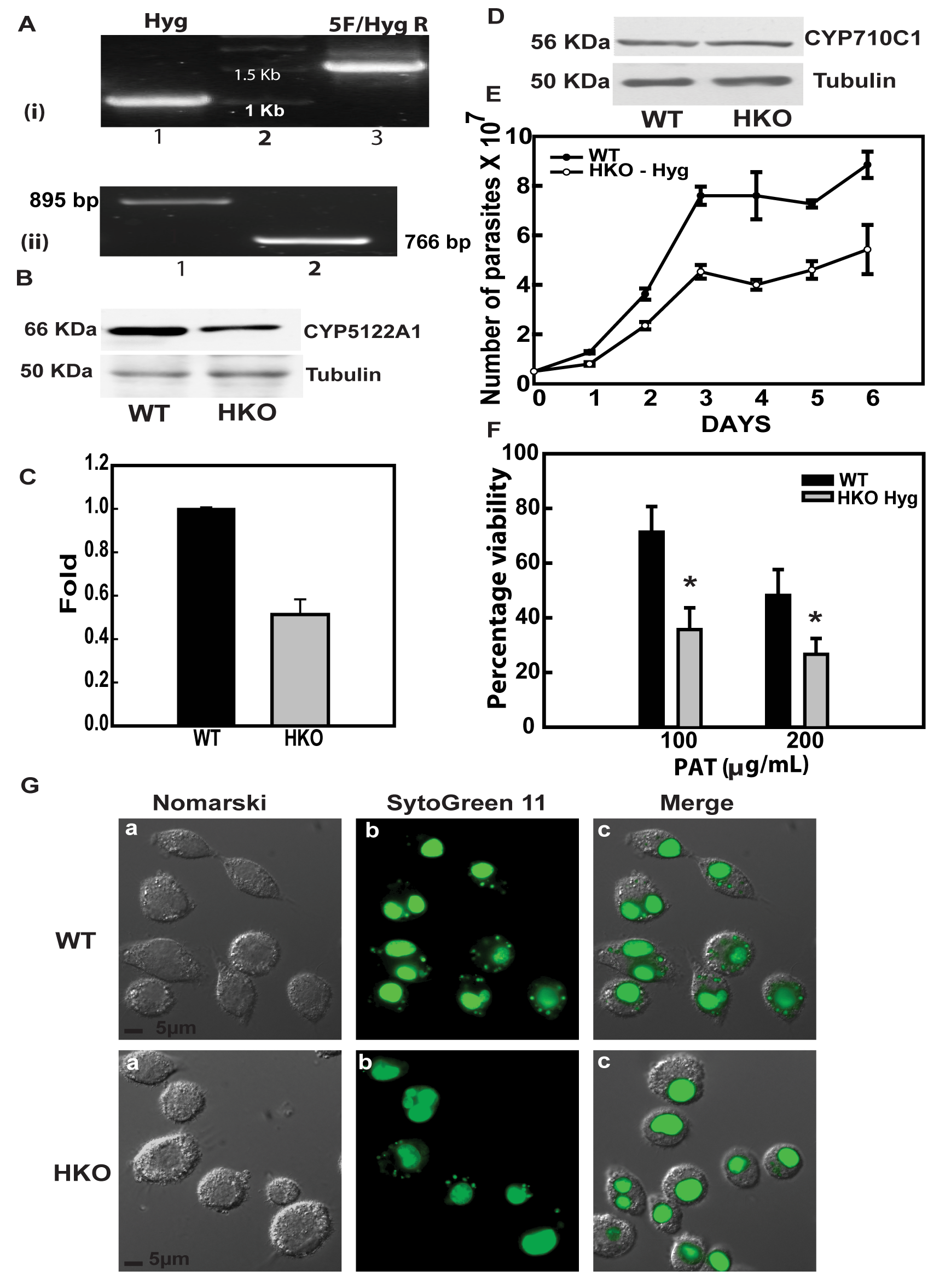

Supplement: Figure S4 — Analysis of pBSK+CYP5122A1-Hyg HKO. A: (i) Insertion of allelic replacement construct into the genomic DNA was confirmed by PCR for hygromycin and knockout-hygromycin construct using primers that span the 5′ homologous sequence (Forward primer) and the hygromycin gene (Reverse primer). Lane 1: fragment amplified for the hygromycin resistance gene; lane 2: 1 kb DNA ladder; lane 3: fragment amplified for the knock out hygromycin construct. (ii) Confirmation of insertion of the replacement construct at the correct locus was assessed by PCR. Lane 1: amplicons generated from HKO genomic DNA as template with primers F2/P2 indicating the presence of an intact CYP5122A1 allele (primer positions indicated in schematic in Fig. 4A); Lane 2: amplicons generated from HKO-Hyg genomic DNA as template with primers F2/HI indicating the presence of insertion of hygromycin resistance ORF at the correct locus. B: Western blot analysis of cell lysates from WT and HKO parasites with anti-CYP5122A1 antibody showing the decreased level of CYP5122A1protein in the HKOs. Loading was normalized with Tubulin–α (50 kDa). C: Bar graph representing averaged densitometric analysis of immunoblots represented in B, mean± SE, n = 3. D: Western blot analysis of cell lysates from WT and HKO parasites using anti-CYP710C1 antibody showing equal levels of the CYP710C in the WT and the HKO parasites. Loading was normalized with tubulin-α (50 kDa). E: Comparison of growth and survival of WT and HKO parasites in vitro over a period of 6 days in culture. Mean ± SE, n = 3. F: Bar graph showing percentage viability of WT and HKO parasites in response to treatment with PAT. Note the increased susceptibility of HKOs as compared with WT. Mean ± SE, n = 4; * P≤0.05. G: Photomicrographs of macrophage infected with WT and HKO parasites stained with Syto 11 Green fluorescent nucleic acid stain. a: nomarski image; b: Syto 11 Green stained image; c: merge of a and b. Scale, 5 µm. (TIF) [file pone.0025273.s004.tif]

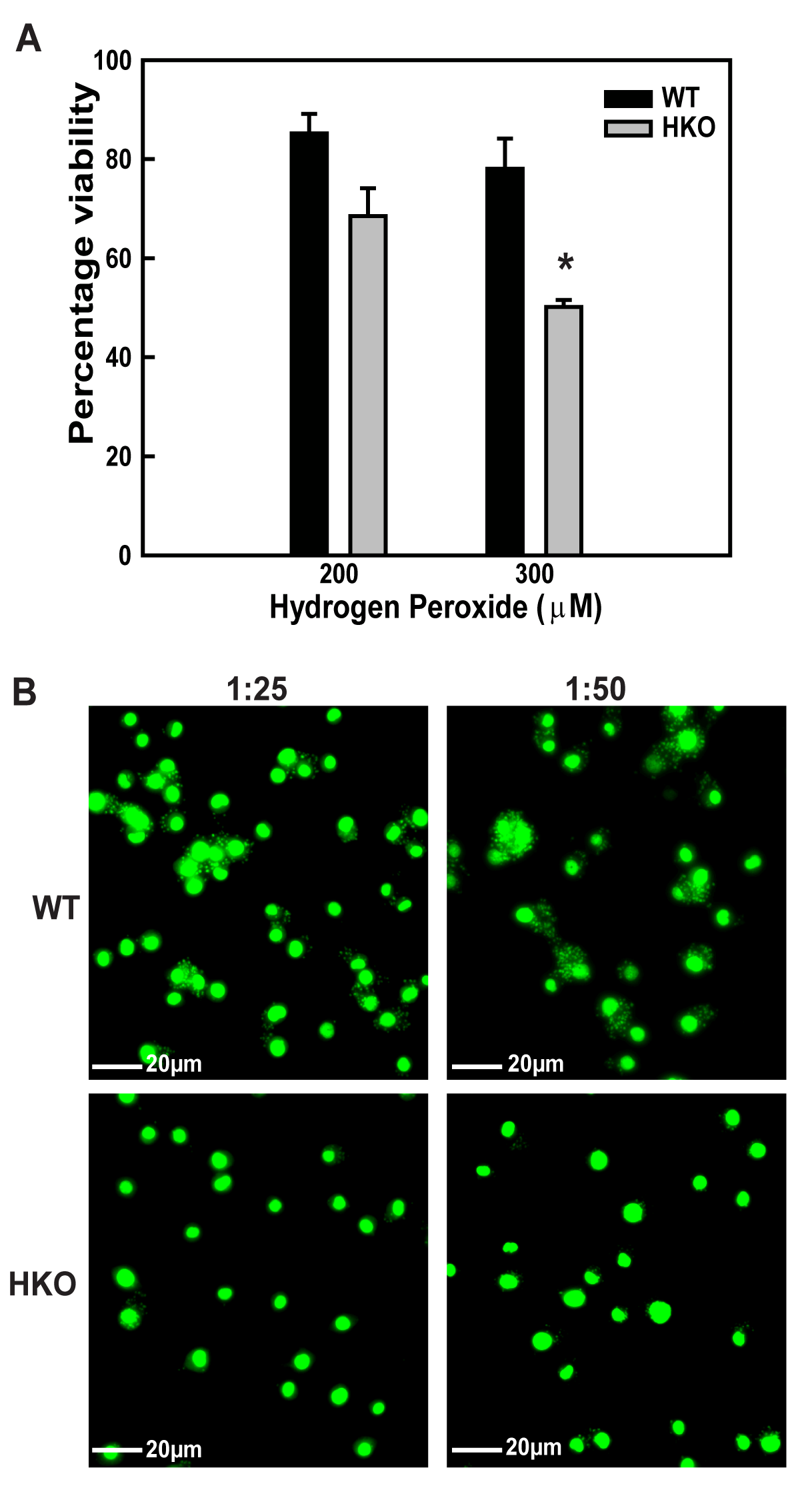

Supplement: Figure S5 — A: Bar graph shows percentage viability of WT and HKO parasites in response to treatment with H2O2. Mean ± SE, n = 4; * P≤0.05. B: Photomicrographs of J774A.1 macrophages infected with WT and HKO L. donovani parasites at multiplicity of infection at 1∶25 and 1∶50, stained with Syto 11 Green. Scale, 20 µm. (TIF) [file pone.0025273.s005.tif]

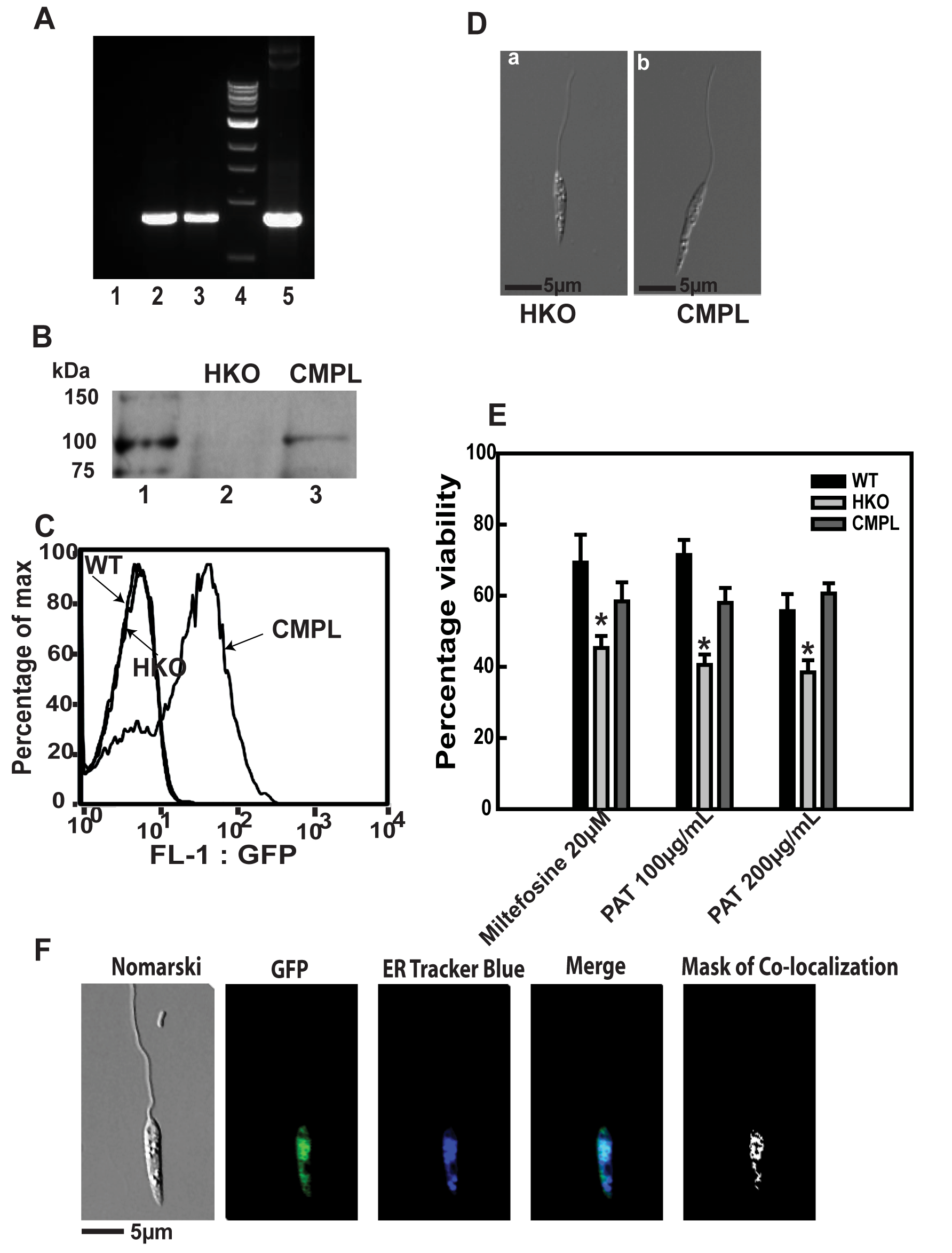

Supplement: Figure S6 — Effects of complementation of CYP5122A1 in HKOs. A: Agarose gel (1.5%) showing expression of GFP-CYP5122A1 fusion mRNA (GFP Forward primer & CYP5122A1 internal primer) in 2 clonally selected strains of CYP5122A1 complemented parasites. Lane 1: negative control; lane 2 & 3: fusion product amplified from c-DNA prepared from complemented parasites; lane 4: 1 kb DNA Ladder; lane 5: Positive control amplified using pXG-GFP+2CYP5122A1 plasmid as the template. B: Western blot analysis of lysates of HKO and CMPL cells probed for the fusion protein show the presence of GFP-CYP5122A1 protein at expected molecular weight of approximately 96KDa in lysates of complemented parasites. C: Flow cytometric analysis of complemented parasites. D. Photomicrographs of HKO (a) and complemented (b) parasites. Scale, 5 µm. E: Bar graph showing percentage viability of WT, HKO and complemented parasites in response to treatment with miltefosine and PAT. Mean ± SE, n = 4; * P≤0.05. F: Photomicrographs of CMPL promastigotes stained with anti-CYP5122A1 antibody and ER-specific markers, ER Tracker Blue-White DPX. Scale, 5 µm. (TIF) [file pone.0025273.s006.tif]

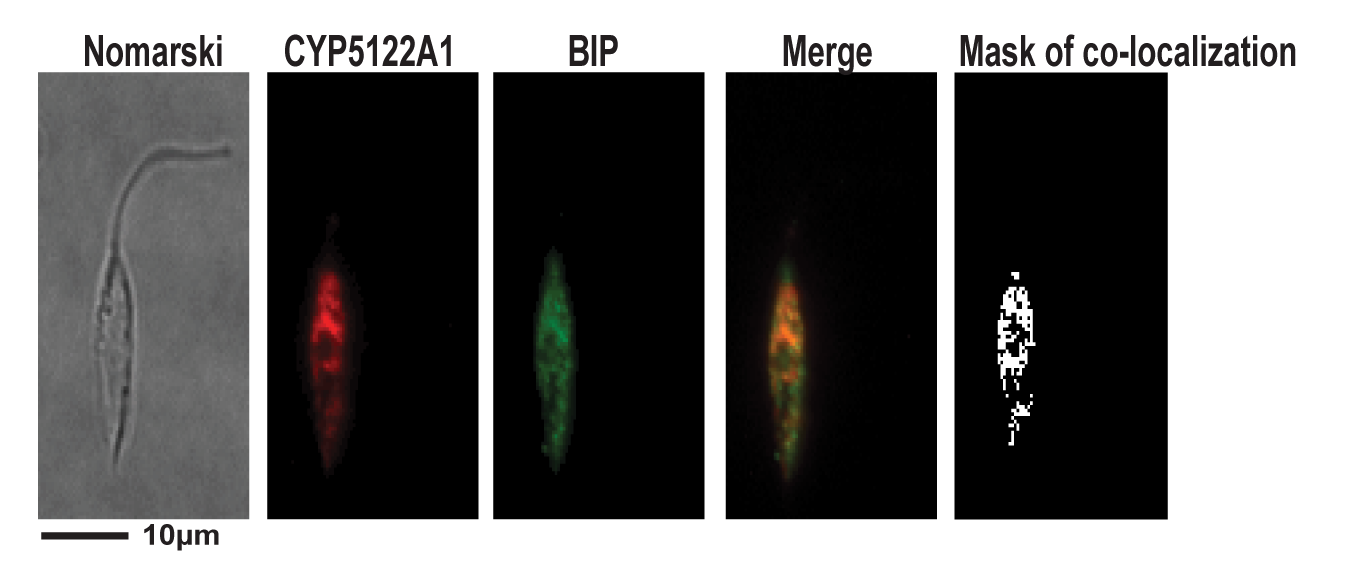

Supplement: Figure S7 — Subcellular localization of CYP5122A1 in metacyclic parasites Photomicrographs of Leishmania donovani metacyclic parasites stained with anti-CYP5122A1 antibody and ER-specific marker, BIP. Scale represents 10 µm. (TIF) [file pone.0025273.s007.tif]
